# Biological Activity of Biomarkers Associated With Metastasis in Osteosarcoma Cell Lines

**DOI:** 10.1002/cam4.70391

**Published:** 2025-03-13

**Authors:** Nidia Ednita Beltrán‐Hernández, Luis Cardenas, Verónica Jimenez‐Jacinto, Leticia Vega‐Alvarado, Heriberto Manuel Rivera

**Affiliations:** ^1^ Instituto de Biotecnología, Universidad Nacional Autónoma de México Cuernavaca Morelos Mexico; ^2^ Departamento de Biología Molecular de Plantas, Instituto de Biotecnología, Universidad Nacional Autónoma de México Cuernavaca Morelos Mexico; ^3^ Unidad Universitaria de Secuenciación Masiva y Bioinformática, Instituto de Biotecnología, Universidad Nacional Autónoma de México Cuernavaca Morelos Mexico; ^4^ Instituto de Ciencias Aplicadas y Tecnología, Universidad Nacional Autónoma de México Coyoacán Ciudad de México Mexico; ^5^ Universidad Autónoma del Estado de Morelos, Facultad de Medicina Cuernavaca Morelos Mexico

**Keywords:** cancer biomarkers, metastasis, osteosarcoma, transcriptome

## Abstract

**Introduction:**

Osteosarcoma, a highly aggressive bone cancer primarily affecting children and young adults, remains a significant challenge in clinical oncology. Metastasis stands as the primary cause of mortality in osteosarcoma patients. However, the mechanisms driving this process remain incompletely understood. Clarifying the molecular pathways involved in metastasis is essential for enhancing patient prognoses and facilitating the development of targeted therapeutic strategies.

**Methods:**

RNA sequencing (RNA‐Seq) analysis was employed to compare three conditions, hFOB1.19 versus Saos‐2, hFOB1.19 versus SJSA‐1, and Saos‐2 versus SJSA‐1, involving non‐cancer osteoblasts (hFOB1.19) and highly metastatic osteosarcoma cell lines (Saos‐2 and SJSA‐1). Additionally, ENA datasets of RNA‐Seq from osteosarcoma biopsies were included. Differentially expressed genes (DEGs) were identified and analyzed through enrichment pathway analysis and protein–protein interaction (PPI) networks. Additionally, for gene candidates, a biochemical evaluation was performed.

**Results:**

DEGs associated with biological functions pertinent to migration, invasion, and metastasis in osteosarcoma were identified. Notably, matrix metalloproteinase‐2 (MMP‐2) emerged as a promising candidate. Both canonical or full‐length (FL‐*mmp‐2*) and N‐terminal truncated (NTT‐*mmp‐2*) isoforms were discerned in biopsies. Moreover, MMP‐2's activity was characterized in cell lines. Additionally, mRNA expression of voltage‐gated sodium channels (Na_V_s) and voltage‐gated potassium channels (K_V_s) was detected, and their functional expression was validated using patch clamp techniques. Evaluation of cell line migration and invasion capacities revealed their reduction in the presence of ion channel blockers (TTX and TEA) and MMP inhibitor (GM6001).

**Conclusions:**

The gene functional enrichment analysis of DEGs enabled the identification of interaction networks in osteosarcoma, thereby revealing potential biomarkers. Moreover, the elucidated co‐participation of TTX‐sensitive Na_V_s and MMP‐2 in facilitating migration and invasion suggests their suitability as novel prognostic biomarkers for osteosarcoma. Additionally, this study introduces a model delineating the potential interaction mechanism among ion channels, MMP‐2, and other crucial factors in the metastatic cascade of osteosarcoma.

## Graphical abstract

Osteosarcoma presents diagnostic and treatment challenges due to its unclear etiology and frequent late‐stage detection. This study aims to identify metastasis biomarkers in osteosarcoma cell lines and biopsies. Through RNA evaluation, a gene functional enrichment analysis of DEGs enabled the identification of interaction networks in osteosarcoma, revealing potential biomarkers. Biochemical analysis elucidates the co‐participation of TTX‐sensitive Na_V_s and MMP‐2 in facilitating migration and invasion, suggesting their suitability as novel prognostic biomarkers. Additionally, this study introduces a model delineating the potential interaction mechanism among ion channels, MMP‐2, and other crucial factors in the metastatic cascade of osteosarcoma.

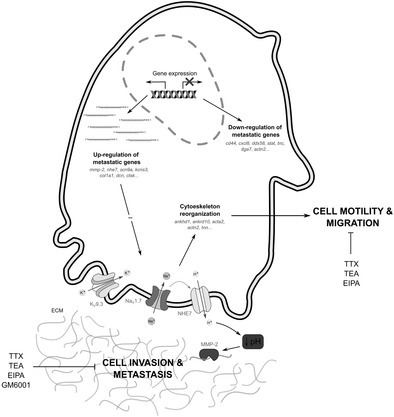

## 1 Introduction

Osteosarcoma is a challenging cancer to diagnose and treat due to its unclear etiology and frequent late‐stage detection. With a global incidence of 8–11 cases per 1,000,000 in children and adolescents, it is considered a rare disease. Despite accounting for only 20% of pediatric malignant tumors, it is the third most common cancer in adolescents, known for its aggressive and metastatic nature. Patients with pulmonary metastasis have a poor 5‐year survival rate of 19%–30%, emphasizing the need for effective therapeutic strategies [1, 2].

Metastasis, the primary cause of cancer‐related deaths, is a complex process involving the spread and colonization of cancer cells in distant sites from the primary tumor. It encompasses multiple sequential steps driven by spatial and temporal factors. The early stages entail cancer cell invasion and migration in the vicinity of the primary tumor [3, 4].

In the context of cancer, cellular proliferation triggers a dynamic transformation of the adjacent extracellular matrix (ECM) through an interplay between the microenvironment and resident cells. This malignancy disrupts tissue organization and modifies cellular behavior due to genetic mutations and epigenetic changes. Tumors are compared to non‐healing wounds, leading to the synthesis and deposition of ECM proteins by recruited fibroblasts, inducing mechanical stress that may prompt cell transdifferentiation [5, 6, 7]. These alterations involve the increased secretion of specific ECM proteins, which disrupt cell adhesion, polarity, and enhance growth factor signaling, fostering tumor progression. The invasion pattern aligns with linearized collagen fibers, indicating their role in facilitating tumor invasion. Cancer cells exhibit the ability to migrate through tissues and breach the adjacent basement membrane. The ECM, composed of various molecules, plays a pivotal role in shaping the tumor microenvironment, involving proteoglycans, glycosaminoglycans, structural proteins, adhesion proteins, and MMPs [7, 8, 9].

MMPs, consistently expressed across diverse tissues, play a pivotal role in various physiological processes, including cellular differentiation, mobility, angiogenesis, apoptosis, and tissue remodeling. However, alterations in MMP expression can result in pathological conditions characterized by tissue destruction, loosening of the ECM, and fibrosis. As proteolytic enzymes, MMPs are essential to the dissolution of ECM components, establishing them as recognized biomarkers, particularly in cancerous pathologies [7, 10]. In cancer development, MMPs orchestrate the degradation of the ECM, triggering a fundamental change in cellular phenotype and facilitating epithelial–mesenchymal transition (EMT). This transition involves the loss of cell polarity and cell‐to‐cell adhesion, enhancing the invasive potential of tumor cells. MMPs, by degrading collagen, expose normally concealed sites in the ECM, enabling integrins to interact with its components. The cancer tumor microenvironment is distinguished by stimulated ECM degradation via MMP activity, linked with the release of local growth factors and angiogenesis [7, 8, 9].

MMP‐2 plays a pivotal role in cancer progression, invasion, and metastasis, influencing various stages of the metastatic cascade, including intravasation, extravasation, and pre‐metastatic niche remodeling. The enzyme's capacity to cleave and degrade the ECM and basement membrane underscores its significance in tumor development. Its activity is regulated at multiple levels, involving transcriptional control, post‐translational modification, secretion, zymogen activation, inhibitor modulation, and protein degradation. In cancer, MMP2 is frequently overexpressed, and elevated protein levels correlate with adverse prognostic factors, such as poor differentiation, metastasis to secondary organs, and resistance to chemotherapy [11, 12].

The intricate interplay among MMPs, the ECM, and ECM molecules is indispensable for the cancer progression. Notably, Na_V_1.5 plays a significant role in enhancing cell invasiveness within this intricate framework. It achieves this by activating Na^+^/H^+^ exchanger‐1 (NHE‐1), leading to perimembrane space acidification and the subsequent activation of ECM proteases, ultimately resulting in ECM degradation. Moreover, Na_V_1.5 instigates changes in F‐actin polymerization through Src kinase activation, thereby fostering cell invasion [13, 14].

In a related context, the heterologous expression of Na_V_1.6 in C33A cervical carcinoma (CeCa) cells induces a substantial fivefold increase in invasiveness, an effect that can be reversed by TTX. The heightened activity of MMP‐2 observed in this context implies that Na_V_1.6 modulates CeCa invasiveness through MMP‐2 activation [15]. These findings underscore the complex molecular mechanisms involving MMPs and Na_V_ channels, shedding light on their collaborative role in cancer cell invasion and metastasis.

Recognized as crucial players in the early stages of carcinogenesis, MMPs have been proposed as therapeutic targets in various malignancies. Despite challenges, particularly in advanced cancer stages, investigating the connections between ECM degradation induced by MMPs and various signaling pathways involved in tumor development holds promise for insights that could mitigate life‐threatening metastasis, especially in osteosarcoma.

RNA‐Seq provides a valuable approach for understanding the molecular aspects of cancer, including drug resistance, heterogeneity, and biomarker discovery. It enables accurate assessment and analysis of complex biological systems, facilitating the development of targeted therapeutic strategies for cancer treatment [16]. For instance, toll‐like Receptor 7 (TLR7) was identified as a DEG in osteosarcoma samples using RNA‐Seq. Its expression was significantly associated with the prognosis of osteosarcoma. As expected, silencing TLR7 led to a decrease in the migratory and invasive capabilities of osteosarcoma cell lines, suggesting it as a potential target for metastasis treatment [17].

The objective of this study is to identify metastasis biomarkers in osteosarcoma cell lines and biopsies. Through RNA‐Seq analysis, we examined gene expression patterns in hFOB1.19, Saos‐2, and SJSA‐1 cells. Comparative analysis revealed DEGs associated with invasion and metastasis in osteosarcoma. Additionally, RNA‐Seq datasets from the ENA database were utilized to validate the expression of DEGs in biopsies. Bioinformatic analysis pinpointed MMP‐2 as a biomarker for osteosarcoma metastasis, with both FL‐ *mmp* *‐2* and NTT‐*mmp* *‐2* isoforms identified in biopsies. Furthermore, the activity of MMP‐2, Na_V_s, and K_V_s was assessed. Migration and invasion capacities were diminished in the presence of TEA, TTX, and GM6001 in osteosarcoma cells. These findings provide preceding evidence suggesting the cooperative involvement of Na_V_s and MMPs in facilitating migration and invasion in osteosarcoma.

## 2 Materials and Methods

### 2.1 Culture of Cell Lines

The cell lines Saos‐2 (ATCC HTB‐85; RRID: CVCL_0548) and SJSA‐1 (ATCC CRL‐2098; RRID: CVCL_1697) were used as metastatic model for osteosarcoma, while as non‐metastatic osteoblast controls the hFOB1.19 (ATCC 11372; RRID: CVCL_3708) cell line was used. All cell lines were maintained according to the manufacturer's instructions with 1% of penicillin–streptomycin (Biowest L0010) at 37°C in a CO_2_ incubator.

### 2.2 RNA Library Construction and Sequencing

Short‐read cDNA sequencing library strategy was employed to analyze mRNA expression using the Illumina Genome Analyzer GAIIx platform. The extraction of total RNA, mRNA enrichment, RNA fragmentation, cDNA synthesis, cDNA fragmentation, amplification, and sequencing were performed according to the manufacturer's recommendations. Briefly, to sequence the polyadenylated fraction of RNA, total RNA was isolated from hFOB1.19, Saos‐2, and SJSA‐1. The quality of total RNA was analyzed by RNA integrity number (RIN), and samples with a high RIN value (> 8) were used (Table S1). For each sequencing library 2 μm of total RNA was utilized. Two biological replicates for each cell line were sequenced, and the single‐end RNA‐Seq model was employed. The data that support the findings of this study are openly available in ENA EMBL‐EBI (https://www.ebi.ac.uk/ena/browser/home) with Project ID PRJEB65286.

### 2.3 Mapping, Transcript Assembly, and Expression Level Estimation

The reads obtained by sequencing were cleaned using Trim Galore! Software (Babraham Bioinformatics‐Babraham Institute). This involved eliminating adapters and sequences with a PHRED value < 20. The reads conserved were mapped to the human reference genome (Homo sapiens GRCh38.p12 RefSeq: GCF_000001405.33 from NCBI) and quantified with Kallisto‐Pachter Lab [18]. The quantification values were reported in counts per million (CPM). Genes with expression levels at CPM values ≥ 2 were considered for expression analysis. Differential expression analysis was conducted using edgeR [19], comparing Saos‐2 versus SJSA‐1, hFOB1.19 versus Saos‐2, and hFOB1.19 versus SJSA‐1. DEGs were filtered according to the threshold of false discovery rate (FDR) ≤ 0.05 and, fold change (logFC) value ≥ 2 (considering the housekeeping genes values~1.5 logFC) [20, 21]. The volcano maps of DEGs were plotted using ggplot R package [22]. Complexheatmap library in R package was performed to draw heatmaps [23].

### 2.4 RNA‐Seq Data Analysis of Osteosarcoma Biopsies

Additionally, RNA‐Seq data from osteosarcoma biopsies were obtained from the European Nucleotide Archive (ENA; https://www.ebi.ac.uk/ena/browser/home), under project IDs PRJNA698672 and PRJNA51801. The PRJNA698672 project consists of mRNA sequencing from both tumor (osteosarcoma) and adjacent normal bone tissue (At) biopsies of five male patients aged 11–20 years. In contrast, the PRJNA518013 project includes samples from osteosarcoma tumors and normal tissue from 10 male and female patients, with no age data provided. For the expression analysis of biopsies, the osteosarcoma samples were compared to the normal tissue samples from each project. The biopsy data underwent the same statistical treatment as described previously. This treatment involved considering genes with CPM values ≥ 2, identifying DEGs with FDR values ≤ 0.05, and logFC values ≥ 2.

### 2.5 Gene Functional Enrichment Analysis

To explore the biological function and cellular processes related to DEGs, we conduce a functional enrichment analysis of the Kyoto Encyclopedia of Genes and Genomes (KEGG) with the R pathfindR package [24]. Gene‐ID, logFC, and *p*‐value were used as input data frame. For the active subnetwork‐oriented enrichment analysis, an adjusted *p* < 0.05 was considered.

### 2.6 PPI Network Construction

The DEGs were used to construct PPI networks. The Search Tool for the Retrieval of Interacting Genes/Proteins (STRING) database was used to predict the interactions among protein products of DEGs, considering a high confidence score ≥ 0.7 [25]. Gephi and igraph R package [26, 27] were used to reconstruct, analyze, and visualize the network. The topological parameters used to evaluate the PPI networks were betweenness centrality (BC), degree, and hub nodes.

BC was employed as a parameter to visualize the network. Briefly, it measures how much a gene node acts as an intermediary between other nodes. This metric is calculated based on the number of shortest paths that pass through the target gene node. This score is moderated by the total number of shortest paths existing between any couple of gen nodes of the graph. The target gene would have a high betweenness centrality if it appears in many shortest paths. Degree or connectivity refers to the number of edges incident on a particular node. Nodes with a large number of connections are considered hubs. According to Equation (1), the hub value was calculated as the mean plus the standard deviation of the degree distribution. This equation was adapted from Rakshit et al. [28].

(1) Hub=mean+SDof the degree distribution

### 2.7 Gelatin Zymography

To evaluate the activity on the MMPs, a gelatin zymography approach was performed. This method is based on analyzing the digestion of a substrate such as gelatin added to polyacrylamide gel. Firstly, 1.6 × 10^6^ cells were seeded in 24‐well plates and incubated for 24 h in complete media (10% fetal bovine serum or FBS), the media were removed and washed with PBS twice. Media with 1% FBS was added to the cell culture and used as a problem sample. While media with 1% FBS and 100 ng/mL of phorbol 12, 13‐dibutyrate (PDBu) were used as a positive control. For negative control media with 1% FBS and 100 ng/mL of PDBu without cells were used. The cell culture was incubated for 40 h; after incubation 500 ul of the supernatant of each cell line and condition was added to Amicon ultra centrifuge filters (Merk Millipore) and centrifugated for 20 min at 15,000 × *g*. The total protein of supernatant concentrate was quantified by Bradford Protein Assay, as standard bovine serum albumin (BSA) was used. Total protein samples (0.1 μg) were mixed with sample buffer (2.5% SDS, 4‐μg/mL phenol blue, 40% glycerol) under non‐reducing conditions. Samples were separated by electrophoresis on 10% polyacrylamide gels containing 1ug/ml gelatin. After electrophoresis, the gels were washed three times for 25 min with 2.5% triton X‐100 and incubated in activity buffer (50 mM Tris–HCl, pH 7.4; and 5 mM CaCl_2_) at 37°C for 40 h. The gels were incubated in stained solution containing 0.25% Coomassie Brilliant Blue G‐250, 10% acetic acid, and 30% methanol. Proteolytic activity was visualized by destaining in methanol‐acetic acid. Quantification of bands intensities corresponding to~95 and~72 KDa molecular weight ladder were performed by ImageJ software [29].

### 2.8 Migration and Invasion Assays

Transwell permeable support chamber was performed to determine the migration and invasion ability of osteosarcoma cell lines [15, 30]. Briefly, 6 × 10^4^ cells of each cell line were seeded in the inserts using culture media with 1% FBS in the absence or presence of each cell line were seeded in the inserts using culture media with 1% FBS in the absence or presence of channel blockers: TTX 1 μM (Sigma‐Aldrich, St. Louis, MO), Tetraethylammonium (TEA) 10 mM (Sigma‐Aldrich); protease inhibitors E‐64 25 μM (Millipore), GM6001 100 μM (Millipore), and the NHE‐specific inhibitor: 5‐(N‐etil‐N‐isopropyl)amiloride (EIPA) 10 μM (Sigma‐Aldrich). The inserts were immersed in the lower chamber which contained 800 μL of enriched culture medium with 10% FBS. For the cell transmembrane invasion assay, all the steps were carried out similarly to those in the migration assay except for the Matrigel coating. hFOB1.19 was used as no cancer condition control. After incubation at 37°C for 24 h, the filters were removed. The cells adhering to the lower surface were fixed and stained with DAPI. To image the cells, three randomly selected fields were taken in each well at 10 × magnification by Olympus FCV1000 Upright BX61WI confocal microscope and counted in three independent experiments.

### 2.9 Electrophysiology

Electrophysiological recordings were carried out after 1 and not more than 8 h after seeding. Cells were replaced from the recording chamber every 50 min. The macroscopic activity of Na_V_ and K_V_ currents were examined using the whole‐cell configuration of the patch‐clamp technique [31]. Current recordings were obtained at 21°C using an Axopatch 200B amplifier, a Digidata1322a converter, and pCLAMP 9.4 software (Molecular Devices; San Jose, CA). Currents were digitalized at 10–20 kHz, after 5 kHz analog filtering. Whole‐cell series resistance and cell capacitance were estimated from optimal cancelation of the capacitive transients with the built‐in circuitry of the amplifier and in some cases was compensated electrically by 60%–70%. Cells were bathed in a solution containing the following composition (in mM): for sodium currents, 164 NaCl, 2 CaCl_2_, 1 MgCl_2_, and 10 HEPES‐NaOH (pH 7.4); for potassium currents, 158 NaCl, 10 TEA‐Cl, 2 CaCl_2_, and 10 HEPES‐NaOH (pH 7.4). Borosilicate glass pipettes (WPI Inc., Sarasota, FL) with resistances of 2–3 MΩ were filled with an internal solution containing (in mM): 100 CsCl, 30 NaF, 2 CaCl_2_, 1 MgCl_2_, 10 EGTA, and 10 HEPES‐CsOH (pH 7.3). TTX (Sigma‐Aldrich, St. Louis, MO) was dissolved in the external solution at a final concentration of 1 μM.

Na_V_s currents were evoked by 16‐ms depolarizing pulses to potentials from −80 to +80 mV, in 10‐mV increments applied every 10 s from a holding potential of −100 mV. Whereas for I_K+_ depolarizing pulses lasted 100 or 200 ms. Current recordings were analyzed using Clampfit software (Molecular Devices) and plotted with Prism 7.0 software.

### 2.10 Quantitative RT‐PCR

To investigate whether Na_V_s subunits are related to osteosarcoma, their mRNA expression was verified through a quantitative RT‐PCR (RT‐qPCR). Total RNA from the hFOB1.19, Saos‐2, and SJSA‐1 were purified with TRI Reagent (Zymo Research) and cleaned with Direct‐zol RNA miniprep kit (Zymo Research). The cDNA library was generated from 2 μg of total RNA in a 20 μL of High Capacity cDNA Reverse Transcription Kit (Applied Biosystems). For the PCR reaction was used TaqMan Gene Expression Master Mix (Invitrogen, cat: 4369016), 500 nM of each 5′ and 3′ primer, and 250 nM of the probe (Table S1), the RT‐qPCR was performed in the Roche Light Cycler Nano with one step of 2 min at 50°C and 10 min at 95°C for enzyme activation, and then 40 cycles with denaturation at 95°C for 15 s, probe annealing at 69°C for 15 s, and 1 min for primers annealing and extension at 60°C. Negative controls were included using the reaction mix without cDNA, primers, or probe. All RT‐qPCR reactions were performed in triplicate, and the cycle threshold (Ct) values were averaged. Relative expression FC was calculated using 2^−ΔΔCt^ [32, 33]. Housekeeping Proteasome subunit beta type 2 (*psmb2*) gene was used as an expression control. Amplification primers and probes for RT‐qPCR were designed using the Primer designing tool from NCBI (https://www.ncbi.nlm.nih.gov/tools/primer‐blast/), and by the OligoCalc (http://biotools.nubic.northwestern.edu/OligoCalc.html). The primers Tm were verified by gradient PCR (data not shown).

## 3 Results

### 3.1 Identification of DEGs

Osteoblast (hFOB1.19) and osteosarcoma (Saos‐2 and SJSA‐1) cell lines were used as models for studying osteosarcoma. Total RNA was extracted from each cell line to construct the cDNA, which was then sequenced using Illumina Genome Analyzer GAIIx in a single‐end mode. Two biological replicates were obtained for each cell line. The sequence reads for hFOB1.19, Saos‐2, and SJSA‐1 were 4.8 × 10^7^, 4 × 10^7^, and 5 × 10^7^ respectively.

**FIGURE 1:**
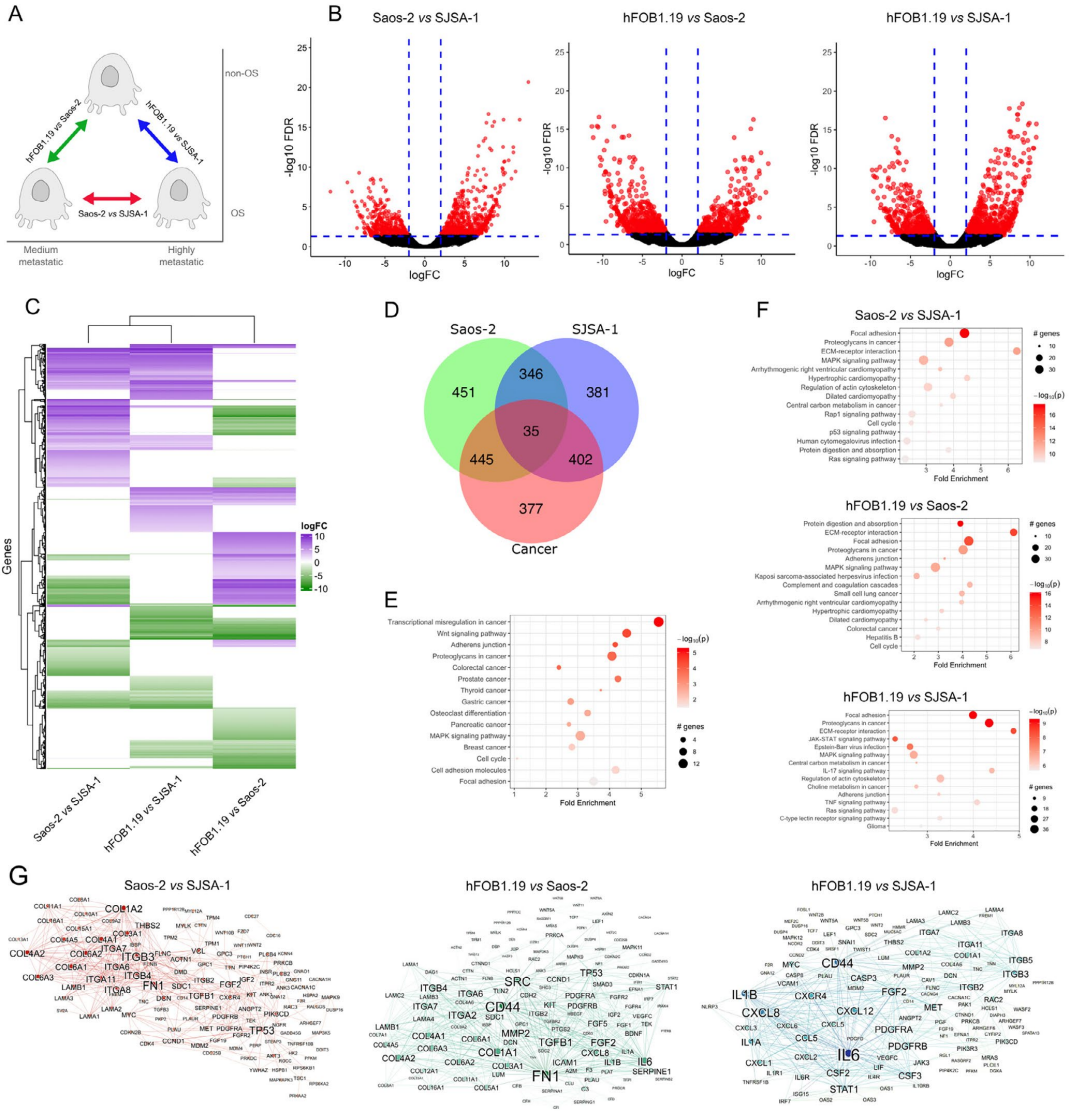
Integrated analysis of DEGs in osteosarcoma cell lines. (A) Three‐way DEGs analysis between non‐osteosarcoma (non‐OS) and osteosarcoma (OS) cells. (B) Volcano plots of DEGs in osteosarcoma cells. Dots represent genes. Dashed lines indicate significance thresholds (logFC > 2; FDR < 0.05). (C) Heatmap and hierarchical clustering of DEGs in each expression analysis, with downregulated genes in green and upregulated genes in magenta. (D) Venn diagrams showing the number of overlapping and unique DEGs for each comparison in osteosarcoma cell lines. (E) Top 15 enrichment KEGG pathways analysis of overlapping DEGs in the Venn diagrams. (F) Top 15 enrichment KEGG pathways analysis for each comparison in osteosarcoma cell lines. (G) PPI network of DEGs identified from the top 15 enrichment pathways in osteosarcoma comparisons. Nodes represent genes, and edges denote interactions, showcasing pivotal molecular entities associated with the highly enriched pathways.

The gene expression profile was assessed through three comparisons: Saos‐2 versus SJSA‐1, hFOB1.19 versus Saos‐2, and hFOB1.19 versus SJSA‐1 (Figure 1A). DEGs were chosen based on criteria: logFC ≥ 2 and FDR ≤ 0.05. In the comparison with both cancer cell lines, 1259 DEGs were identified, with 766 genes (61%) upregulated and 493 genes (39%) downregulated. hFOB1.19 versus Saos‐2 revealed 1277 DEGs, with 577 genes (45.2%) upregulated and 700 (54.8%) downregulated. In hFOB1.19 versus SJSA‐1, 1164 DEGs were found, comprising 680 (58.4%) upregulated and 484 (41.6%) downregulated (Figure 1B). The heat map illustrates the expression pattern of DEGs in the three conducted comparisons. Each row in the heat map represents a gene, while each column represents a sample. This visual analysis clearly reveals the characteristic gene regulation patterns for each comparison, providing a graphical representation of variations in DEG expression under different cellular conditions (Figure 1C).

### 3.2 Enrichment Pathway Analysis of DEGs

Next, we obtained the numbers of upregulated and downregulated genes that were unique or shared between the three comparisons. The number of shared genes across all three conditions was 35, with an additional 1193 genes shared between two of the three comparisons (Figure 1D).

The enrichment pathway analysis of these genes indicated the dysregulation of pathways such as transcriptional misregulation in cancer, adherens junction, proteoglycans in cancer, colorectal cancer, prostate cancer, thyroid cancer, and osteoclast differentiation (Figure 1E). Additionally, pathway enrichment analysis was conducted on DEGs in each comparison. In the Saos‐2 versus SJSA‐1 comparison, the identified DEGs are associated with focal adhesion, proteoglycans in cancer, ECM–receptor interaction, MAPK signaling pathway, regulation of actin cytoskeleton, and central carbon metabolism in cancer. In the hFOB1.19 versus Saos‐2 comparison, the pathways related to DEGs include protein digestion and absorption, ECM–receptor interaction, focal adhesion, proteoglycans in cancer, adherens junction, and the MAPK signaling pathway. Additionally, in the hFOB1.19 versus SJSA‐1 comparison, the identified pathways consist of focal adhesion, proteoglycans in cancer, ECM–receptor interaction, JAK–STAT signaling pathway, MAPK signaling pathway, central carbon metabolism in cancer, and regulation of actin cytoskeleton (Figure 1F).

### 3.3 PPI Network Analysis

We subsequently employed the STRING database to construct PPI networks encompassing the putative proteins encoded by the identified DEGs. A PPI network delineates physical interactions among proteins, with the number of connections (edges) associated with a specific node (protein). Nodes with high connectivity indicate direct interactions with numerous other distinct nodes and are considered crucial hubs within the network, often associated with key biological processes. We generated a separate PPI network for each comparison, employing a high confidence score (≥ 0.7) to filter out PPI networks with low probability or significance. The clustering coefficient, average degree, and hub value are detailed in Table S3, with the degree serving as a parameter for network visualization (Figure 1G). Our hub analysis identified several highly‐ranked nodes commonly in the PPI networks. For example, fibroblast growth factor 2 (FGF2), which plays a crucial role in the regulation of cell survival, cell division, cell differentiation, and cell migration, is a common hub in the three comparisons. Additionally, other hubs commonly identified in the comparisons include integrin alpha and beta (ITGA6, ITGA7, ITGA8, ITGB3, and ITGB4), various members of collagen alpha (COL3A1, COL4A1, COL4A2, COL6A2, and COL6A6), Interleukin‐6 (IL6), CD44 antigen (CD44), Interleukin‐8 (CXCL8), and MMP‐2 (Figure 1G, Figure S1).

### 3.4 MMP‐2 Target Network Analysis

**FIGURE 2:**
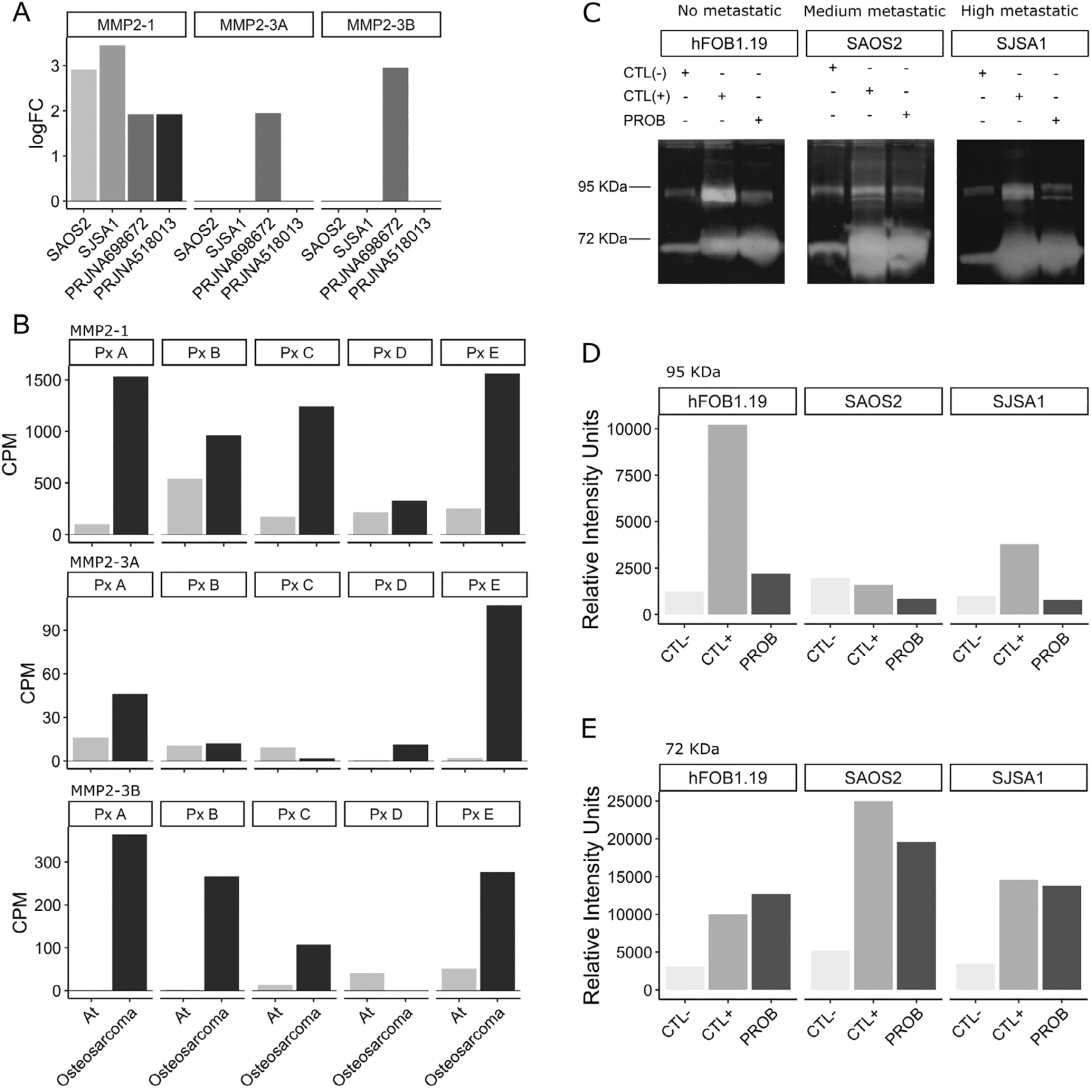
Functional expression of MMPs in osteosarcoma. (A) Significantly different logFC values of *mmp‐2* isoforms in Saos‐2, SJSA‐1, and osteosarcoma biopsies (PRJNA698672 and PRJNA518013 ENA projects). (B) Expression of *mmp‐2* isoforms 1, 3A, and 3B in noncancerous adjacent tissue (At) and osteosarcoma biopsies from five patients (Px A to E). The upper panel shows *mmp‐2* isoform 1 (MMP‐2‐1; NM_004530.5), the middle panel shows *mmp‐2* isoform 3A (MMP‐2‐4; NM_001302509.1), and the bottom panel shows MMP‐2 isoform 3B (MMP‐2‐5; NM_001302510.1) (ENA project PRJNA698672). (C) Gelatin zymography of osteosarcoma cell lines. Lane 1 represented the control condition consisting of media with 1% FBS and 100 ng/mL of PDBu without cells. Lane 2 corresponded to the positive control, involving media with 1% FBS, 100 ng/mL of PDBu, and the cell culture. Lane 3 denoted the sample problem (PROB), with media containing 1% FBS and the cell culture. (D) Quantitative analysis of band intensities was performed for proteins with a molecular weight of 95 kDa. (E) Quantitative analysis of band intensities was carried out for proteins with a molecular weight of 72 kDa. Band intensities were analyzed and compared using ImageJ software.

MMP‐2, which has been identified as a hub in the networks, plays a critical role in cell invasion across different cancer types. Moreover, it exhibits synergistic effects with Na_V_s [15]. The evaluation of the logFC of the *mmp‐2* gene and its isoforms (*mmp‐2‐*1, *3A*, and *3B*) was conducted in cancer cell lines and biopsies of osteosarcoma tumors from the PRJNA698672 and PRJNA518013 projects of the ENA‐EMBL. The PRJNA698672 and PRJNA518013 projects involve mRNA sequencing of both tumor (osteosarcoma) and adjacent normal bone tissue (At) biopsies. For the expression analysis, the osteosarcoma samples were compared with their corresponding normal tissue samples within each project. Interestingly, the FL‐*mmp‐2* was found to be differentially expressed in all four conditions. While the NTT‐*mmp‐2* (denoted in this work as 3A and 3B) exhibited equal or higher expression only in the PRJNA698672 project. It is worth noting that the mean age of the patients in the PRJNA698672 project was 15.4 ± 3.9 years old (Figure 2A).

The assessment of the expression of FL‐*mmp‐2* and NTT‐*mmp‐2* in the biopsies obtained from patients (PxA to PxE) in the PRJNA698672 project demonstrated elevated expression levels in cancer in comparison to adjacent tissue (At). The NTT‐*mmp‐2* 3A isoform exhibited predominantly increased expression in patients A and E, whereas the NTT‐*mmp‐2* 3B isoform revealed elevated expression in the majority of patients (Figure 2B).

### 3.5 Functional Expression of MMPs

To evaluate the proteolytic activity of MMP‐2 in cell cultures, a gelatin zymography assay was performed. The assay detected two MMPs (~72 and~95 kDa), corresponding to FL‐MMP‐2 and MMP‐9, respectively. In the control conditions of all three cell lines, minimal activity from intrinsic MMPs in FBS was observed (Figure 2C, left line; and D). In contrast, the three cell cultures were treated with PDBu to induce the upregulation of MMP expression. Treated samples exhibited a substantial increase in intensity for the ~72 kDa bands, as depicted in the middle line of the gels (Figure 2C–E). Upon comparing the minimal activity of intrinsic MMPs in FBS between the left and right lines, no discernible difference in activity was observed, indicating the absence of activity in the ~95 KDa band in the cell lines (Figure 2C,D).

Nevertheless, a similar upregulated effect was observed in the middle and right lines for the ~72 KDa bands, which corresponded to the positive control and problem samples, respectively. This finding indicates an increase of activity in the relative migration of the ~72 KDa proteins in both the cancer and hFOB1.19 cell lines (Figure 2C,E). These results correlate with the transcript accumulation found in the DEGs experiments and demonstrate that MMP‐2 is differentially expressed and its encoded protein is functional in osteosarcoma cell lines.

### 3.6 The Role of MMP‐2 in Migration and Invasion

**FIGURE 3:**
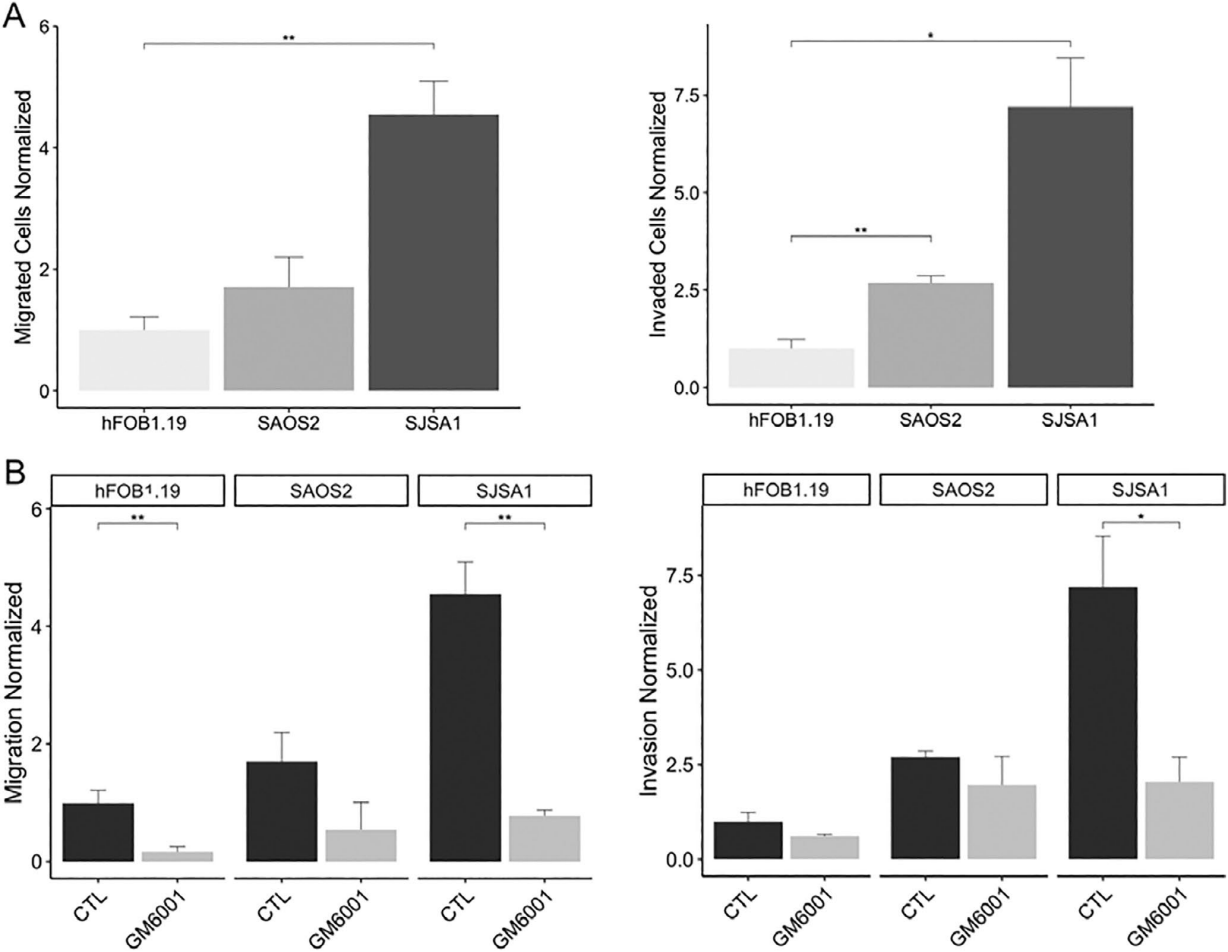
Migration and invasiveness in osteosarcoma cell lines. (A) Evaluation of migratory and invasive capacities of hFOB1.19, Saos‐2, and SJSA‐1. (B) Migration and invasiveness evaluation of osteosarcoma cell lines treated with the MMPs inhibitor GM6001 (100 μM). Migrated and invaded cells were statistically analyzed (*n* = 3 individual experiments). The groups were analyzed with an ANOVA test. *Significantly different *p* < 0.05, **p < 0.01, ***p < 0.001. All values are means ± SE (error bars).

Also, transwell assays were conducted to assess the influence of MMP‐2 on migration and invasion in osteosarcoma cell lines. Our findings have revealed distinct numbers of migratory and invasive cells across the different cell lines studied. Specifically, SJSA‐1 exhibited a significantly higher number of migratory cells (178 ± 21.39) in comparison with both Saos‐2 (66.67 ± 19.4) and hFOB1.19 (39.11 ± 8.46). Likewise, SJSA‐1 demonstrated a greater number of invasive cells (56 ± 9.66) compared to Saos‐2 (20.89 ± 1.35) and hFOB1.19 (7.78 ± 1.84) (Figure 3A; Figures S2–S5). Saos‐2 exhibits moderate migration and invasion capabilities, surpassing hFOB1.19 cells by approximately two times in migratory capacity and three times in invasive capacity. In contrast, SJSA‐1 cells show the highest migratory and invasive capacities, exhibiting approximately 4.5 times more migratory and seven times more invasive cells compared to hFOB1.19 cells (Figure 3A; Figures S2–S5).

Cell migration and invasion assays were conducted using the MMP inhibitor GM6001. Interestingly, a significant decrease in migratory cells was observed in both hFOB1.19 and SJSA‐1 cell lines, while no significant difference was seen in the Saos‐2 cell line. Similarly, the invasion assay showed a reduction in the number of invasive cells. However, no significant difference was observed in the hFOB1.19 and Saos‐2 cell lines. In contrast, there was a notable decrease (> 60%) in the number of invasive cells in the SJSA‐1 cell line, suggesting a potential involvement of MMPs in the migration and invasion phenotype observed in these osteosarcoma cell lines (Figure 3B, Figures S2–S5).

### 3.7 Functional Expression of Na_V_s

**FIGURE 4:**
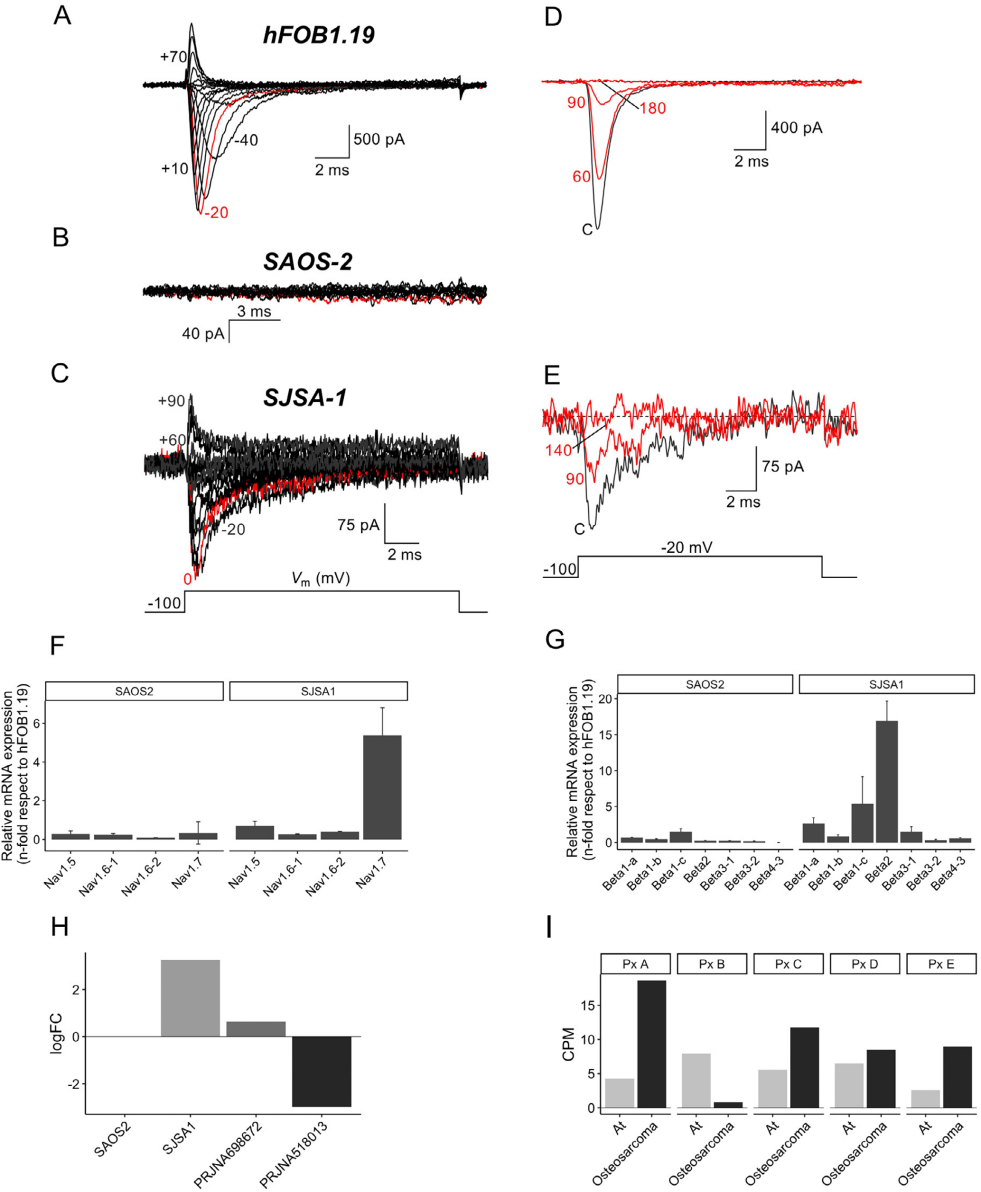
Functional expression of Na_V_s in osteosarcoma. Representative whole‐cell sodium currents recorded from hFOB1.19 (A) and SJSA‐1 (C) cell lines, whereas sodium currents were totally absent in Saos‐2 (B). Red traces indicates where the maximal inward peak current was obtained for each cell line. (D, E) hFOB1.19 and SJSA‐1 sodium currents were fully blocked by 1 μM of TTX. Recordings were obtained at −20 mV in the absence (black traces) and after several tens of seconds (as indicated) in the presence of the TTX (red traces). (F) Expression levels of Na_V_s genes evaluated by RT‐qPCR analysis, (G) expression levels of subunits genes in Saos‐2 and SJSA‐1 versus hFOB1.19. The fold‐change ratio was calculated with 2^−ΔΔCt^. (H) Significantly logFC values of Na_V_1.7 in Saos‐2, SJSA‐1, and osteosarcoma biopsies (ENA projects: PRJNA698672 and PRJNA518013). (I) Na_V_1.7 expression in noncancerous adjacent tissue (At) and osteosarcoma biopsies in five patients (Px A to E from the ENA project PRJNA698672).

To explore the functional significance of Na_V_s and their synergistic interaction with MMPs in osteosarcoma cells, we conducted whole‐cell patch clamp experiments on hFOB1.19, Saos‐2, and SJSA‐1 cell lines (Figure 4A–E). The results revealed the absence of I_Na+_ in Saos‐2 cells (Figure 4B), whereas hFOB1.19 and SJSA‐1 cells exhibited typical I_Na+_ (Figure 4A,C). Despite the flat morphology of these cells, we were able to record the currents in hFOB1.19 and SJSA‐1 but not in Saos‐2. Furthermore, the observed currents were sensitive to the presence of 1 μM of TTX, confirming the functional expression of Na_V_ α‐subunits in osteosarcoma cell lines (Figure 4D,E). Additionally, we confirmed the functional expression of K_V_s in the plasma membrane of all three cell lines, as evidenced by the biophysical hallmarks of I_K+_ in Figure S6. Notably, no calcium currents were detected in any of the cell lines.

The results of the patch clamp experiments sparked our curiosity, prompting us to delve into the mRNA expression of Na_V_ subunits through RT‐qPCR analysis. It confirmed that there was no differential mRNA expression of α and β Na_V_ subunits in Saos‐2 cells. However, the Na_V_1.7 α‐subunit was significantly overexpressed by fivefold in SJSA‐1 cells (Figure 4F). Moreover, we observed overexpression of β1a, β1c, and β2 subunits (2.63, 5.41, and 12.21 folds, respectively) (Figure 4G), and the relative mRNA expression of Na_V_s was consistent with RNA‐Seq results (Figure 4H,I and Figure S7).

The mRNA expression of Na_V_s subunits was verified using RT‐qPCR. It confirmed that there was no differential mRNA expression of α and β Na_V_ subunits in Saos‐2 cells. However, the Na_V_1.7 α‐subunit was significantly overexpressed by fivefold in SJSA‐1 cells (Figure 4F). Moreover, we observed overexpression of β1a, β1c, and β2 subunits (2.63, 5.41, and 12.21 folds, respectively) (Figure 4G), and the relative mRNA expression of Na_V_s exhibited consistency with the RNA‐Seq results (Figure 4H,I). The expression of Na_V_1.7 was not detected in Saos‐2 cells or in the biopsy samples from the PRJNA518013 project. However, an observable increase in expression was found in SJSA‐1 cells and in the biopsies from the PRJNA698672 project. To assess Na_V_1.7 expression on an individual level in the PRJNA698672 project, we evaluated its expression for each patient. This analysis revealed an upregulation in cancerous tissue compared to adjacent normal bone tissue (At) (Figure 4I).

In addition to the electrophysiology analysis, the present study also evaluated the functional role of Na_V_s, K_V_s, and NHEs in cell migration and invasion through in vitro assays. To achieve this, cell migration and invasion assays were conducted in the presence of blockers of Na_V_s (TTX 1 μM), K_V_s (TEA 20 mM), and NHE‐specific inhibitor (EIPA 10 μM).

**FIGURE 5:**
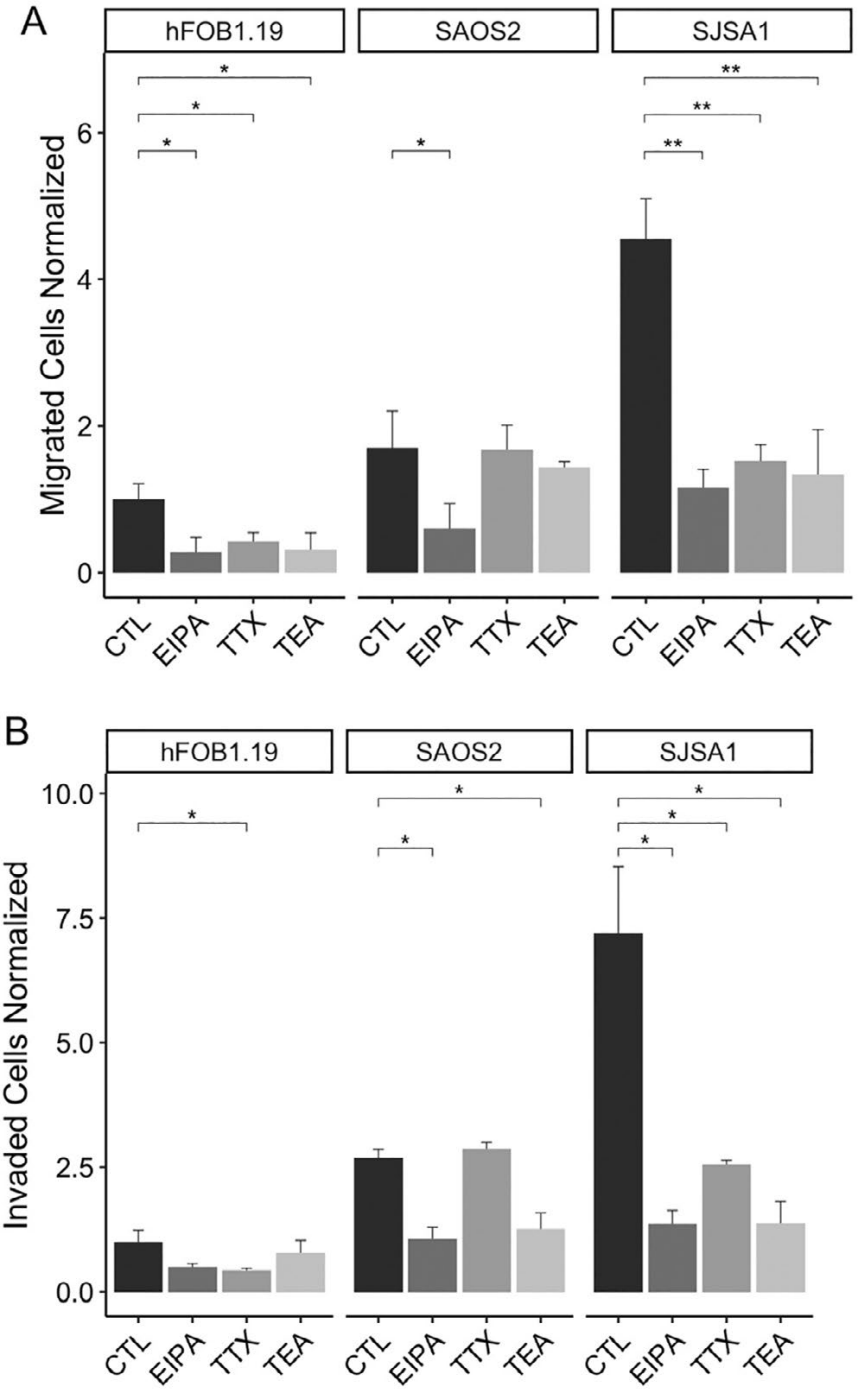
Pharmacological effect in migration and invasion in osteosarcoma cell lines. Migration (A) and invasion (B) evaluation of osteosarcoma cell lines treated with NHE specific inhibitor (EIPA 10 μM), K_V_s blocker (TEA 10 mM), and Na_V_s blocker (TTX 1 μM). Migrated and invaded cells were statistically analyzed (*n* = 3 individual experiments). The groups were analyzed with ANOVA test. *Significantly different *p* < 0.05, **p < 0.01, ***p < 0.001. All values are means ± SE (error bars).

Our findings demonstrated a significant decrease in migratory cells of hFOB1.19 and SJSA‐1 cell lines when treated with all three blockers, while only the NHE blocker had an effect on Saos‐2 (Figure 5A). In invasiveness assays, all three blockers reduced the number of cells in SJSA‐1, while EIPA and TEA showed an effect in Saos‐2. Additionally, exposure to TTX resulted in a reduction in hFOB1.19 (Figure 5B). Notably, no effect was observed in Saos‐2 with TTX treatment, suggesting the absence of Na_V_ expression and currents in this cell line.

## 4 Discussion

In this study, we identified DEGs in three distinct comparisons involving osteosarcoma cell lines (Saos‐2 versus SJSA‐1, hFOB1.19 versus Saos‐2, and hFOB1.19 versus SJSA‐1). The enrichment pathway analysis of these DEGs revealed their involvement in pathways such as focal adhesion, proteoglycans in cancer, ECM–receptor interaction, MAPK signaling pathway, regulation of actin cytoskeleton, adherens junction, and ECM–receptor interaction. These pathways have been previously reported as crucial regulators in osteosarcoma metastasis [34, 35].

The DEGs identified in the pathway enrichment analysis were utilized to construct the PPI networks, encompassing the putative proteins encoded by all DEGs associated with cancer pathways. However, PPI networks are subject to limitations, including reliance on the quality of input data and the potential for undetected or mispredicted interactions. Nevertheless, our stringent statistical analysis of the differential expression data ensured their reliability and accuracy [25, 36, 37, 38]. As a result, STRING emerged as an invaluable tool for investigating PPI networks and generating novel in osteosarcoma biology.

The topological examination of the networks generated in this study facilitated the identification of hubs, which represent highly connected and central nodes within the network. This observation provides insights into their pivotal role in regulating protein interactions. Notable hubs identified in the Saos‐2 versus SJSA‐1 comparison include FN1, ITGB3, TP53, COL1A2, ITGA8, COL6A1‐3, and FGF2. Meanwhile, hubs such as FN1, CD44, SRC, COL1A1, COL4A1, COL4A2, ITGB4, ITGA6‐7, intercellular adhesion molecule (ICAM)‐1, FGF2, and MMP‐2 were identified in the hFOB1.19 versus Saos‐2 comparison. Finally, in the hFOB1.19 versus SJSA‐1 comparison, hubs such as IL6, CD44, FGF2, STAT1, ITGB2‐3, TIGB5, MET, CSF2‐3, and MMP‐2 were identified.

For instance, FGF‐2 emerged as a hub consistently identified across all three comparisons. FGF‐2 is a versatile growth factor with pivotal roles in embryonic development, cell migration, and proliferation. Overexpression of FGF‐2 is associated with increased cell proliferation in various cancers, including lung, breast, gastric, prostate, and melanoma [39, 40, 41]. Our analysis revealed several key interconnected nodes associated with FGF‐2, including TGFB1, TGFB3, WNT, and MAPKs. FGF signaling plays a critical role in osteoblastogenesis through its interactions with various pathways. Specifically, FGF‐2 positively interacts with TGFβ to regulate osteoblast proliferation, modulating TGFβ synthesis at different stages of osteoblast maturation. Additionally, FGF signaling interacts with Wnt/β‐catenin signaling, either positively or negatively [41, 42, 43]. In *Fgf2* knockout mice, Wnt gene expression is reduced, leading to impaired osteoblast differentiation. However, exogenous FGF‐2 can restore Wnt/β‐catenin signaling and osteoblast differentiation, underscoring the crucial role of FGF signaling in promoting osteogenesis through the Wnt/β–catenin pathway [44].

Several studies have identified a relationship between FGF‐2 and MMP‐2. Kim et al. [45] demonstrated that FGF‐2, in combination with fucoidan, promotes endothelial cell proliferation and angiogenesis, while concurrently increasing MMP‐2 activity—potentially involving the p38, JNK, and MMP‐2 signaling pathways. Although the co‐expression of FGF‐2 and MMP‐2 has been documented, the precise mechanisms underlying their interaction remain unclear and require further investigation [45, 46, 47, 48, 49]. Additionally, Fortino et al. [50] observed Na_V_ activation in periodontal ligament stem cells following treatment with EGF and FGF‐2, suggesting a potential link between these growth factors and Na_V_s, which also warrants further exploration [50].

The proposed mechanism of metastasis in osteosarcoma highlights the identification of various collagen types as key hubs. Disruptions in collagen deposition or degradation have significant consequences for ECM homeostasis, affecting the primary functional attributes of the matrix. In the context of tumor progression, dynamic transformations of the ECM, driven by continuous interactions between the microenvironment and resident cells, result in increased secretion of fibronectin and Collagens I, III, and IV. These alterations, marked by enhanced deposition of matrix proteins, contribute to the disruption of cell–cell adhesion, cell polarity, and the potentiation of growth factor signaling, ultimately promoting tumor progression [5, 9].

Additionally, hubs such as SRC, STAT1, IL6, FN1, CD44, IL1B, MMP‐2, ITGB3, ITGB4, and COL1A1 have demonstrated participation in pathways associated with metastasis, invasion, migration, and EMT in osteosarcoma and various cancers, implying their involvement in the regulation of these pathological processes [51, 52, 53].

PosthumaDeBoer et al. [54] and Jerez et al. [55] conducted a proteomic analyses of osteosarcoma cell lines, including Saos‐2 and found specific induced‐proteins. Consistent with these studies, we similarly identified DEGs such as MMP‐2, CD44, COL1A1, COL6A1, FN1, ITGB4, and ITGA2 which were found at the protein level [54, 55]. However, the functional characterization of these identified proteins was not evaluated. Therefore, our study's findings regarding the role of these proteins in migration and invasion are crucial in comprehending the metastatic process in osteosarcoma.

MMPs are key regulators of ECM remodeling and are involved in cancer metastasis. Upregulation of MMPs, including MMP‐2 and ‐9, is associated with aggressive pediatric sarcomas like osteosarcoma, leading to poor prognosis and pulmonary metastasis. MMPs facilitate cancer cell detachment, migration, and invasion through ECM degradation [56, 57, 58]. Recent studies have also highlighted the immune microenvironment panorama in osteosarcoma tumor progression at the single‐cell level [59]. Understanding the role of MMPs and the immune microenvironment can provide valuable insights into osteosarcoma pathogenesis and guide the development of novel therapeutic strategies.

The NTT‐*mmp‐2* isoforms were found to exhibit differential expression in the PRJNA698672 samples. It is noteworthy that the study population consisted of children and adolescents, with a mean age of 15.4 ± 3.9 years. The NTT‐*mmp‐2* isoforms have been implicated in nodulosis–arthropathy–osteolysis (NAO) syndrome, a condition characterized by multiple prominent and painful subcutaneous nodules, extensive osteolysis, arthritis in the hands and feet, and generalized osteoporosis [60]. The cleavage of NTT‐*mmp‐2* from residues 1 to 54 indicates the loss of the signal peptide. In NAO syndrome, this isoform has been reported to exhibit functional loss, particularly in patients from Saudi Arabia. On the other hand, in the context of renal disease, NTT‐*mmp‐2* demonstrates enzymatic activity and intracellular localization, partially within the intramembranous space of the mitochondria [61, 62]. However, its role in cancer remains unreported.

In our study, we successfully identified the gene expression of FL‐*mmp‐2* in osteosarcoma cell lines and biopsies. As FL‐*mmp‐2* has been extensively investigated in various cancer types and is well‐known for its involvement in metastasis, it suggests that FL‐*mmp‐2* similarly play a role in osteosarcoma. However, further research is needed to confirm its role in osteosarcoma.

After identifying the mRNA expression of *mmp‐2*, we proceeded to evaluate the functional expression of gelatinases using gelatin zymography. Our analysis revealed the presence of two active MMPs, with molecular weights of approximately 72 KDa and 95 KDa, corresponding to MMP‐2 and MMP‐9, respectively, in both osteoblastic and cancerous cell lines. Interestingly, Saos‐2 and SJSA‐1 exhibited the highest activity of MMP‐2. Furthermore, this study provides evidence of the functional expression of MMP‐2 and MMP‐9 in hFOB1.19 cells.

Previous studies have reported the activity of MMPs in MG‐63, SJSA‐1, Saos‐2, and U2OS cell lines [63, 64, 65]. Nonetheless, these data highlight a gap in our understanding regarding the specific role of MMPs in these cell lines and their contribution to the molecular mechanisms underlying the migratory and invasive properties of osteosarcoma, and its close relationship with Na_V_s.

The migratory and invasive capacities of hFOB1.19 cells are indicative of their essential roles in bone remodeling, mineralization, and overall bone function [66]. In contrast, the cancerous cells Saos‐2 and SJSA‐1 exhibited significantly increased migratory and invasive activity. Notably, SJSA‐1 displayed a sixfold increase in comparison to hFOB1.19, as previously reported by Lauvrack et al. [67].

A fundamental discovery emerging from this study is the notable reduction observed in these capacities following the administration of inhibitory compounds targeting MMPs. These findings underscore the critical role of MMP‐2 as a molecular target in orchestrating pathophysiological mechanisms, particularly in metastasis, where migration and invasion serve as pivotal pathways driving cancer progression.

Previous studies have suggested the co‐participation of MMP‐2 and Na_V_s in migration and invasion activity in breast and cervical cancer [15, 68]. Upregulation of Na_V_1.7 has been observed in prostate cancer, correlating with increased metastatic potential [69]. In gastric cancer cells, Na_V_1.7 has been linked to I_Na+_ [70]. Na_V_1.7 may affect cancer‐related processes through multiple pathways, including the activation of molecules involved in cell motility, such as PKA, ankyrins, troponins, and gelsolins [71].

We hypothesized that Na_V_1.7 plays a significant role in modulating the function of NHE7, thereby optimizing the pH environment to facilitate MMP‐2 activation and function. These intricate molecular interactions underscore the potential of Na_V_s in orchestrating crucial processes such as migration and invasion. Our study reveals on the complex interplay among Na_V_s, NHE, and MMP‐2, offering valuable insights into cancer progression and suggesting promising avenues for early therapeutic interventions in osteosarcoma.

Similarly, K^+^ channels are the largest and most diverse family of ion channels, and have been extensively studied in cancer‐related cell migration. For example, regulators of tumor cell proliferation and migration, such as K_V_10.1 and K_V_11.1, exhibit upregulated expression that is negatively correlated with patient prognosis [72, 73]. To date, K_V_s have emerged as promising targets in oncology due to their involvement in the regulation of both cell proliferation and apoptosis [74].

In this study, we provide evidence for the up‐regulation of the *kcns3* gene, which encodes K_V_9.3, in the SJSA‐1 cell line. Although the precise role of this potassium channel in cancer remains unclear, our findings demonstrate the recording of potassium channel currents and reveal a significant reduction in migratory and invasive activity following the administration of TEA in both normal and cancer cells. These observations underscore the potential involvement of K_V_9.3 in modulating cellular behaviors associated with migration and invasion within the context of cancer.

Therefore, it is reasonable to propose at the first time for these cell lines that the concurrent activity of Na_V_s and MMPs participates in a mechanism that facilitates migration and invasion. In conditions of upregulation, the functional co‐expression of ionic channels and MMP‐2 enhances the migratory and invasive capacities in cancer. The pronounced difference in functional expression between SJSA‐1 and Saos‐2, indicating that the role of these proteins exerts a significant influence on the progression of the metastatic process.

**FIGURE 6:**
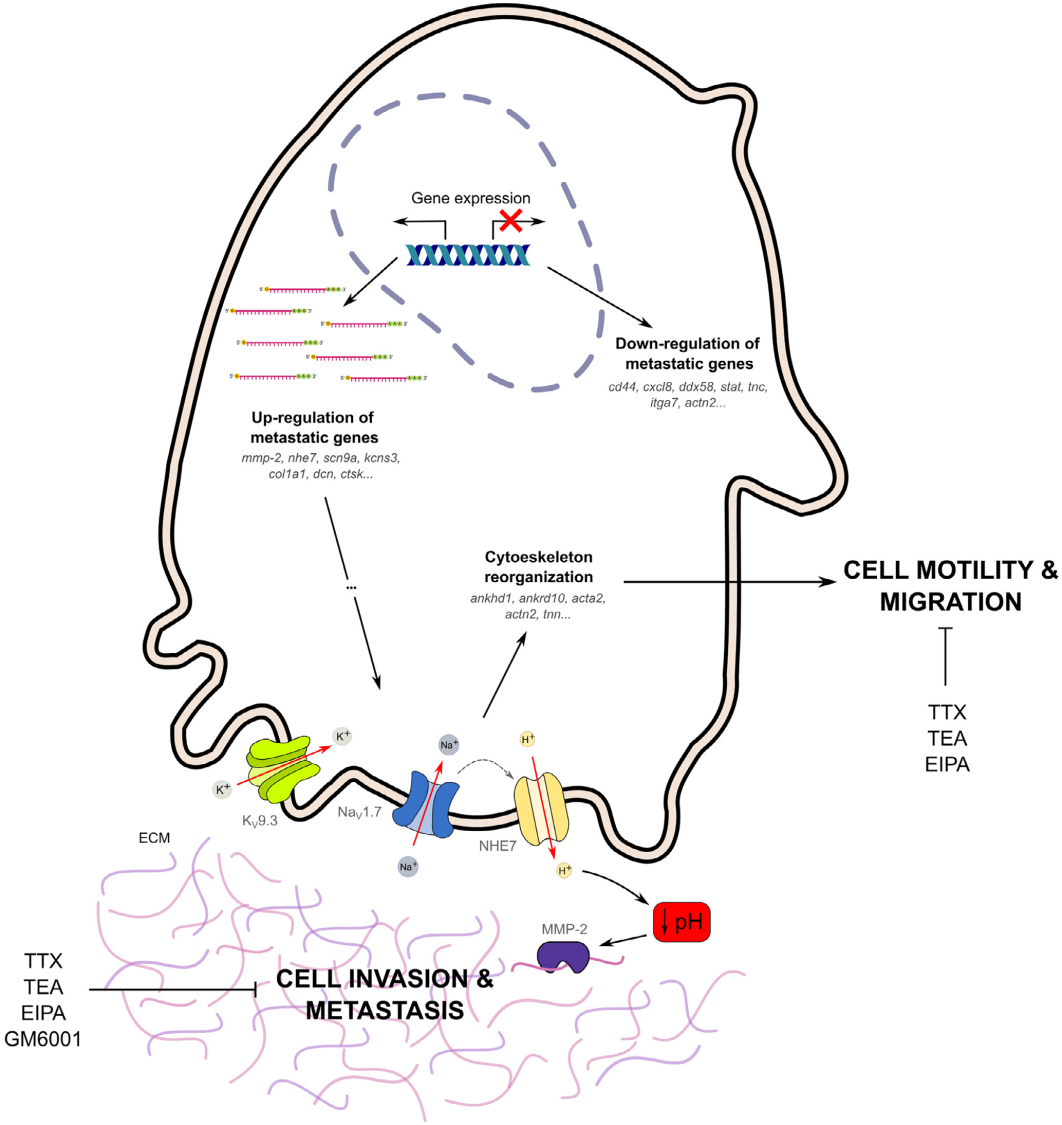
Integrated molecular model of osteosarcoma metastasis. TTX‐sensitive Na_V_s channels co‐express with NHE‐7, promoting proton extrusion and leading to perimembrane acidification. This acidic environment promotes the activity of extracellular proteases, incluiding MMP‐2, consequently leading to ECM degradation. Additionally, the activity of TTX‐sensitive Na_V_s channels, possibly Na_V_1.7, is implicated in modulating actin filaments, thereby facilitating the formation and activity of protrusive structures like invadopodia, consequently enhancing cell motility, migration, and invasion. Furthermore, pharmacological interventions with channel blockers like TTX and TEA, as well as inhibitors such as GM6001 and EIPA targeting MMPs and NHE, respectively, demonstrate a significant reduction in cancer cell migration and invasiveness in vitro.

While the functional activity of MMP‐2 and ionic channels has been documented, particularly in cancer cell proliferation, their specific roles in migration and invasion within the context of osteosarcoma remain understudied. Given the imperative to explore novel avenues in comprehending the metastatic process in osteosarcoma, this study represents the initial endeavor to propose the potential involvement of MMP‐2 and ionic channels in the migratory and invasive behavior of osteosarcoma cells (Figure 6).

Finally, we acknowledge that studying osteosarcoma using cell lines has limitations in fully capturing the tumor microenvironment (TME) and the critical role of interactions between various cell types in tumor progression and treatment resistance. Advances in single‐cell genomics, particularly single‐cell RNA sequencing (scRNA‐Seq), have revealed significant genetic and functional heterogeneity within tumors, identifying distinct cell subpopulations crucial for understanding tumor mechanisms. Within the osteosarcoma TME, both immune cells (e.g., macrophages, NK/T cells, and B cells) and non‐immune cells (e.g., osteoblastic OS cells, endothelial cells, osteoclasts, and cancer‐associated fibroblasts [CAFs]) play vital roles. While immune cells contribute to immune defense, they are often suppressed in the TME. Non‐immune cells are directly involved in tumor growth and metastasis, with CAFs playing a particularly significant role in promoting tumor progression through extracellular matrix remodeling, angiogenesis, and immunosuppression [75, 76, 77, 78, 79]. However, the examination of specific cell types remains a crucial initial step in the identification of genetic and molecular biomarkers. This approach yields valuable insights into cancer mechanisms, establishing a robust foundation for subsequent research that involves more complex models incorporating the diverse cellular interactions within the TME such as patient‐derived xenografts (PDX).

## 5 Conclusion

This study conducted a gene functional enrichment analysis associated with invasion and metastasis in osteosarcoma cell lines, identifying hubs such as FGF2, ITGA6, ITGA7, ITGA8, ITGB3, ITGB4, COL3A1, COL4A1, COL4A2, COL6A2, COL6A6, IL6, CD44, CXCL8, and MMP‐2 as potential biomarkers due to their significance in osteosarcoma networks. Using RNA‐Seq, we identified differential gene expression profiles for *mmp‐2* and *SCN9A*, as well as cell lines and biopsies. Subsequently, the expression of *SCN9A* was validated through RT‐qPCR.

Additionally, TTX‐sensitive Na_V_s were found to exhibit I_Na+_ in both hFOB1.19 and SJSA‐1 cells. We then evaluated the functional roles of TTX‐sensitive Na_V_s and MMP‐2 in migration and invasion, suggesting their potential implication in the migratory and invasive capabilities of SJSA‐1.

Furthermore, I_K+_ were observed in all three cell lines, and their migratory and invasive capacities were reduced in the presence of TEA. However, further investigations are needed to fully understand the role of K_V_s in osteosarcoma hallmarks.

The findings presented here establish a critical foundation for future research focused on identifying novel biomarkers to assess migration and invasion capabilities in osteosarcoma. This approach offers significant insights into cancer mechanisms, providing a robust platform for subsequent studies involving more complex in vivo models.

## Author Contributions

**Heriberto Manuel Rivera:** conceptualization (equal), data curation (equal), formal analysis (equal), funding acquisition (lead), investigation (equal), supervision (lead), writing – original draft (equal), writing – review and editing (equal). **Nidia Ednita Beltrán‐Hernández:** conceptualization (equal), data curation (equal), formal analysis (equal), investigation (equal), writing – original draft (equal), writing – review and editing (equal). **Luis Cardenas Torres:** formal analysis (equal), resources (equal), writing – review and editing (equal). **Verónica Jimenez‐Jacinto:** data curation (equal), formal analysis (equal), writing – review and editing (equal). **Leticia Vega‐Alvarado:** data curation (equal), formal analysis (equal), writing – review and editing (equal).

## Conflicts of Interest

The authors declare no conflicts of interest.

## Supporting information


Table S1.

Figure S1.

## Data Availability

The data generated in this study are available in the ENA‐EMBL (https://www.ebi.ac.uk/ena/browser/home) database under accession number PRJEB65286. The source code is openly accessible in the lbsymt‐developers repository on GitHub at the following link: https://github.com/lbsymt‐developers/Osteosarcoma_DEGs_Analysis.
