# Supplementary material for: Biological Activity of Biomarkers Associated With Metastasis in Osteosarcoma Cell Lines
Source: Cancer Med. 2025 Mar 13;14(6):e70391. doi: 10.1002/cam4.70391 (PMC11904427; doi:10.1002/cam4.70391)
Supplement: Supplementary file 1 — Table S1. [file CAM4-14-e70391-s001.docx]

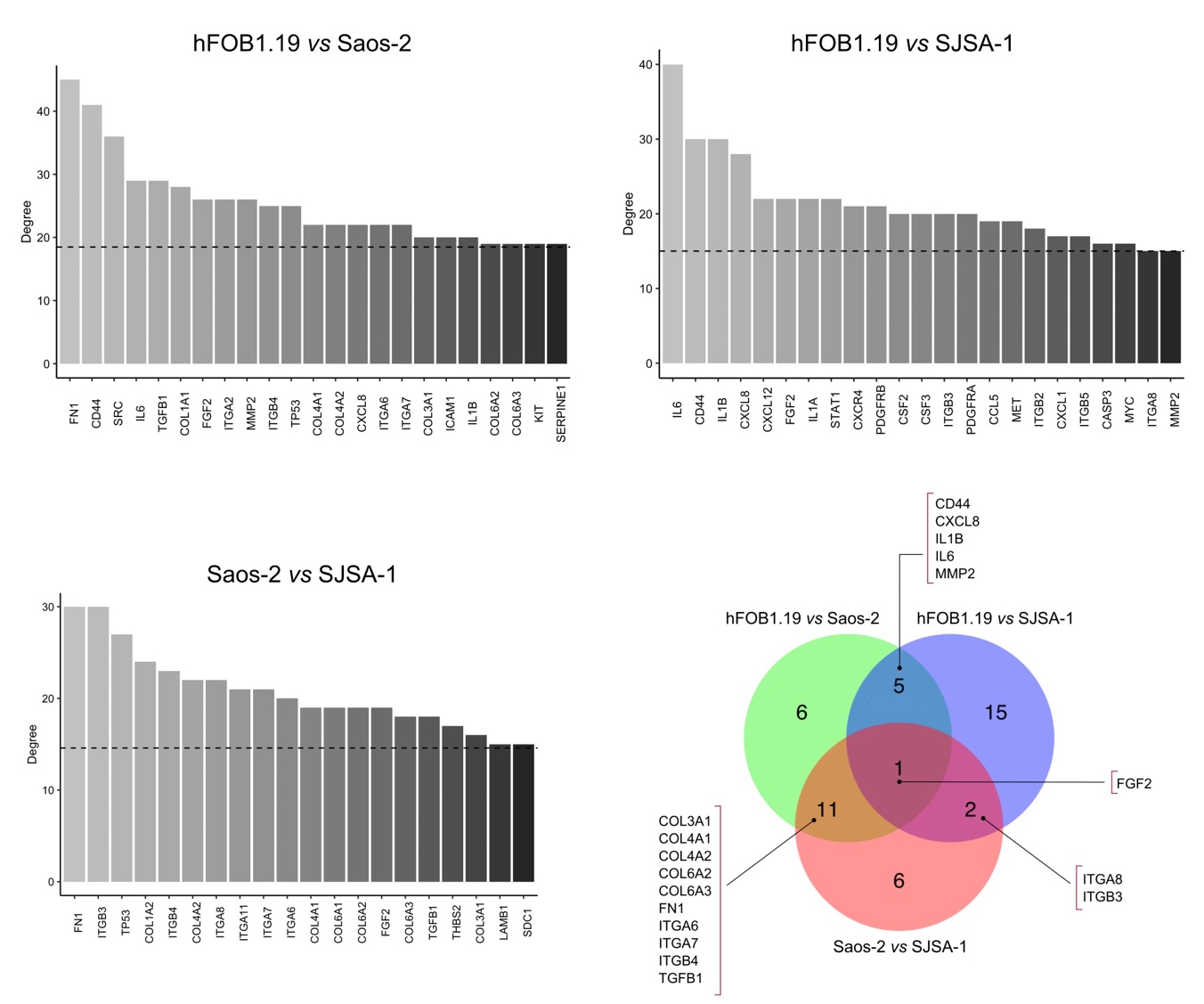


**Figure S1.** For hub identification, a node greater than or equal to the sum of the mean and one standard deviation of the degree distribution was considered a hub (see eq1). The dotted line represents the threshold of hubs in each condition. The hubs were analyzed through a Venn diagram, illustrating the common hubs in the three comparisons.


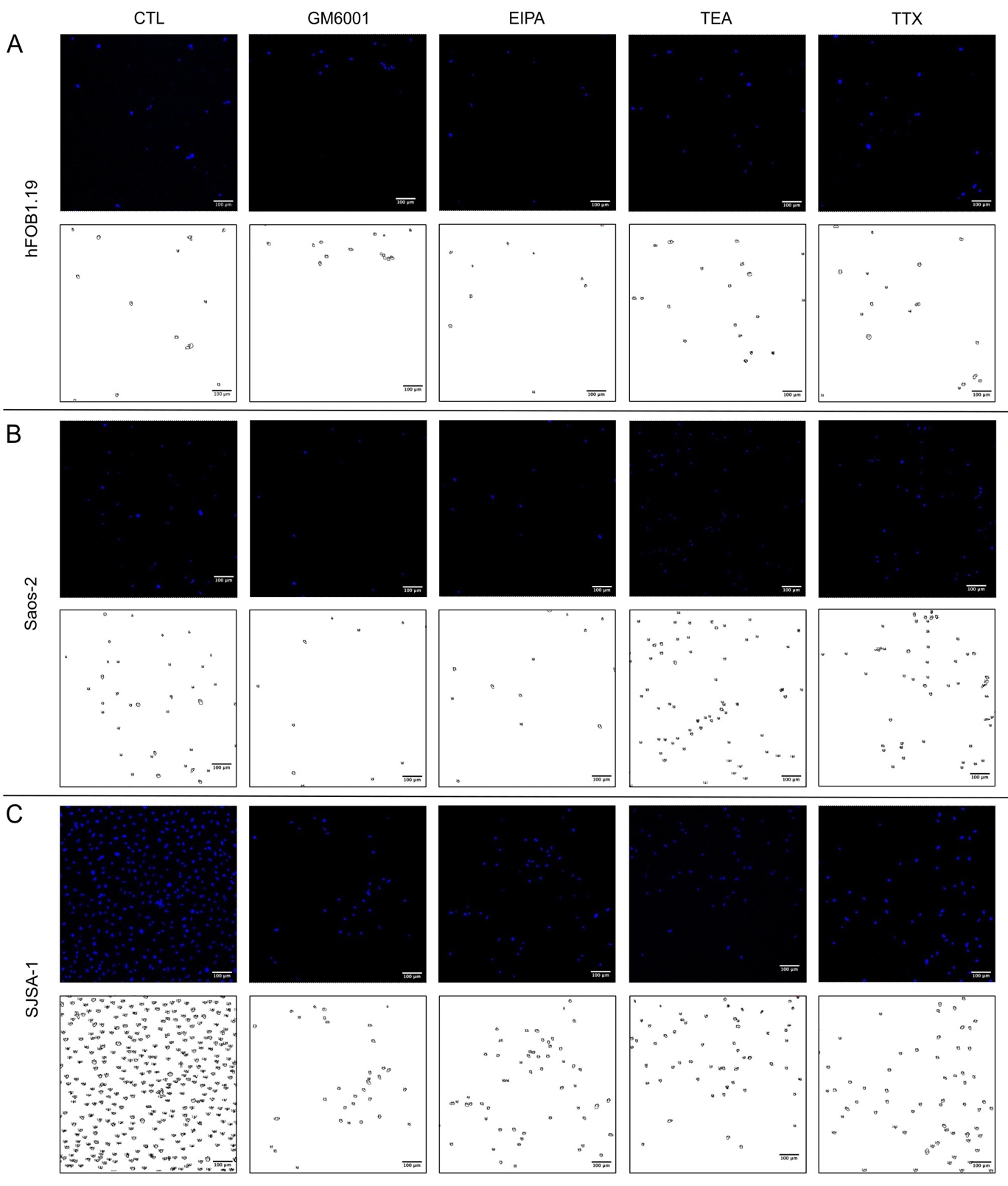


**Figure S2.** Visual representation of the effects of various inhibitors and blockers on cell migration, both in terms of cell distribution and quantification, without any post-processing modifications to the original images. Representative images from migration assays are displayed. The blue filter was applied to visualize cells, and nuclear staining was performed using DAPI (upper panel). Cell count analysis was conducted using the same images. The 'Analyze Particles' function in Fiji was employed to quantify cell numbers, and the results are presented as overlaid count drawings (bottom panel). The panels represent experiments with A) hFOB1.19, B) Saos-2, and C) SJSA-1.


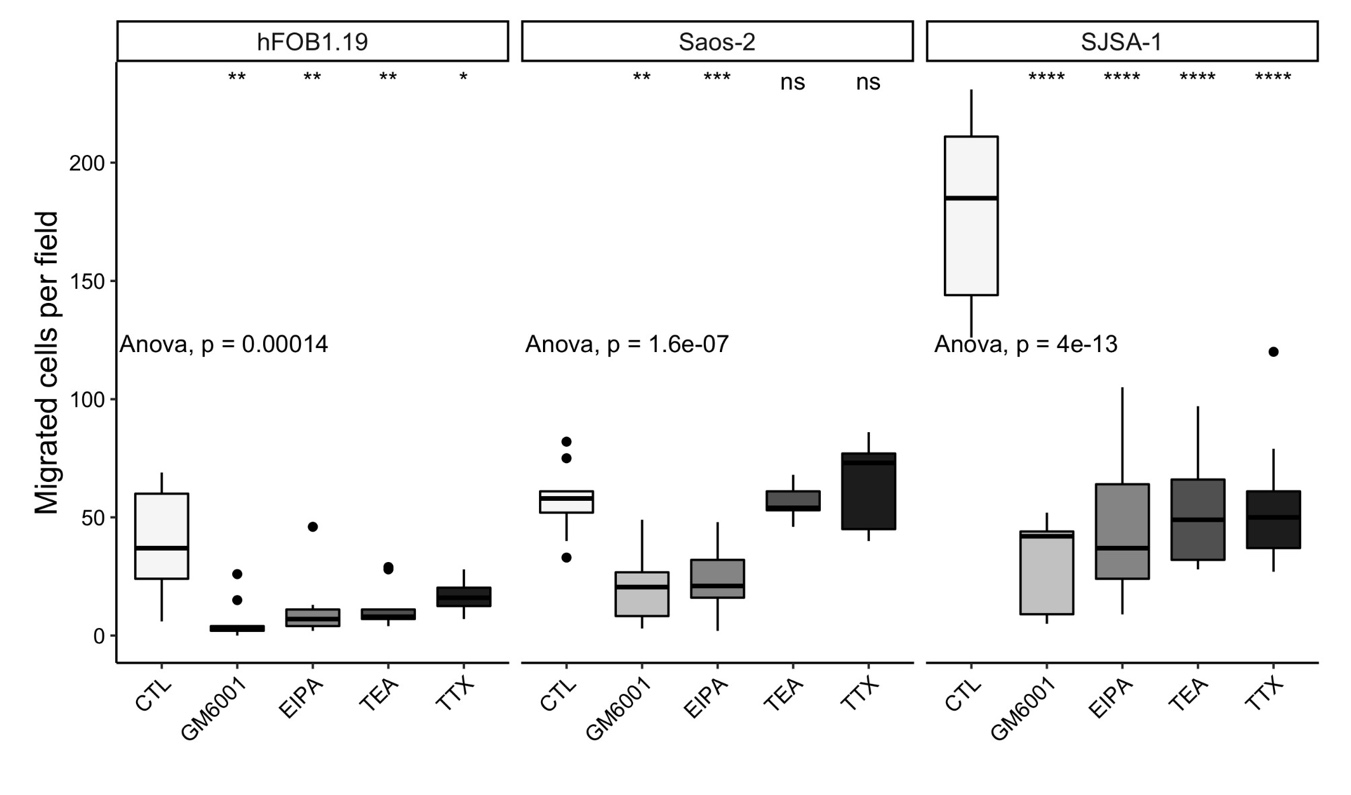


**Figure S3.** Quantification of migratory cells in response to diverse pharmacological treatments. The analysis was based on data from three independent experiments. Statistical analysis using ANOVA was performed to assess the differences among the treatment groups. Significance levels are indicated as follows: ns (not significant, p > 0.05), * (p ≤ 0.05), ** (p ≤ 0.01), *** (p ≤ 0.001), and **** (p ≤ 0.0001).


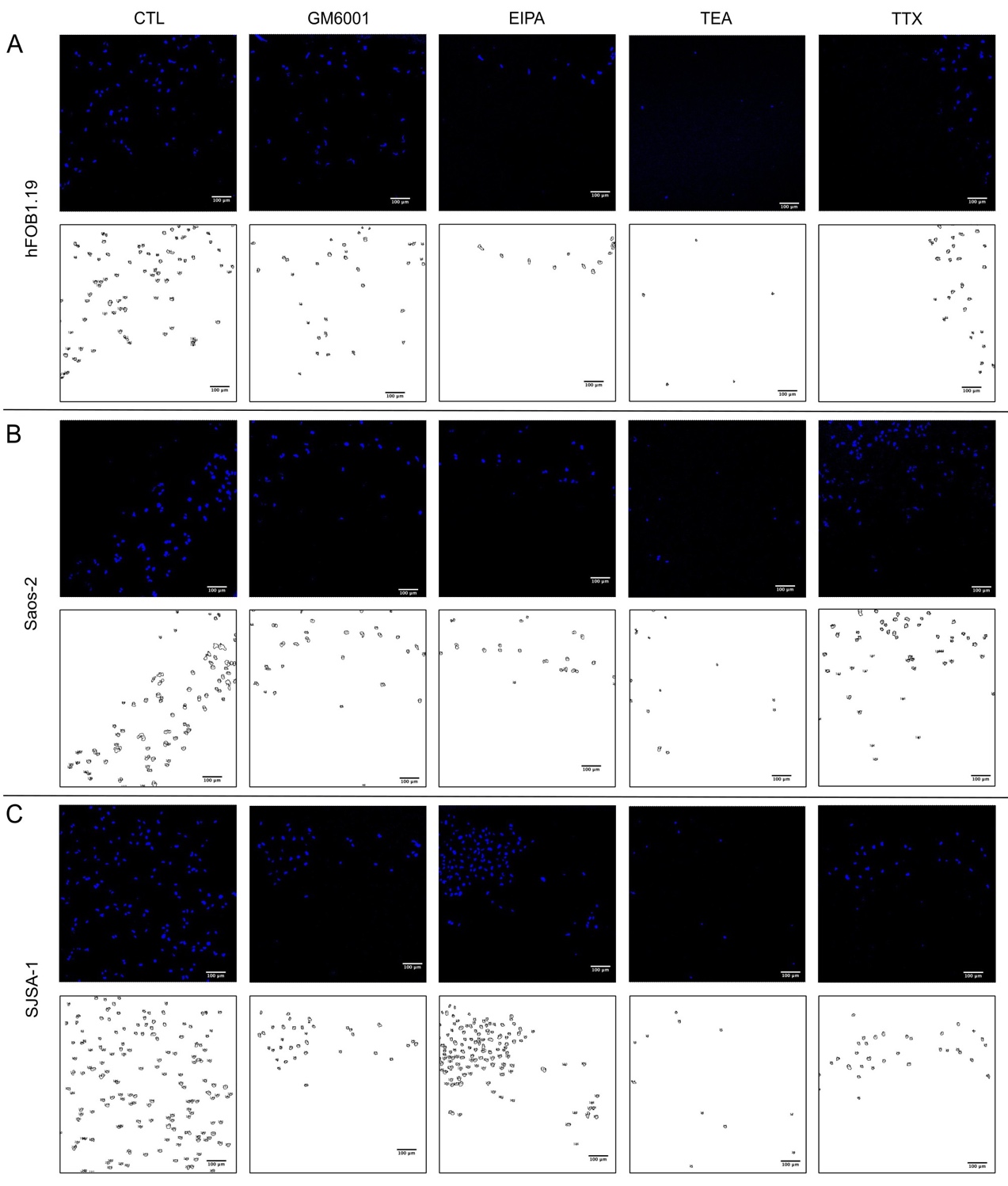


**Figure S4.** Visual representation of cell invasion in the presence of various inhibitors and blockers, including both cell distribution and quantification, without any post-processing modifications to the original images. Illustrative images obtained from invasion assays are presented. The use of a blue filter enhanced cell visualization, while nuclear staining was achieved using DAPI (upper panel). Cell count analysis was conducted using the same set of images. Cell quantification was carried out through the 'Analyze Particles' function in Fiji, and the results are displayed as superimposed count representations (bottom panel). The panels correspond to experiments involving A) hFOB1.19, B) Saos-2, and C) SJSA-1.


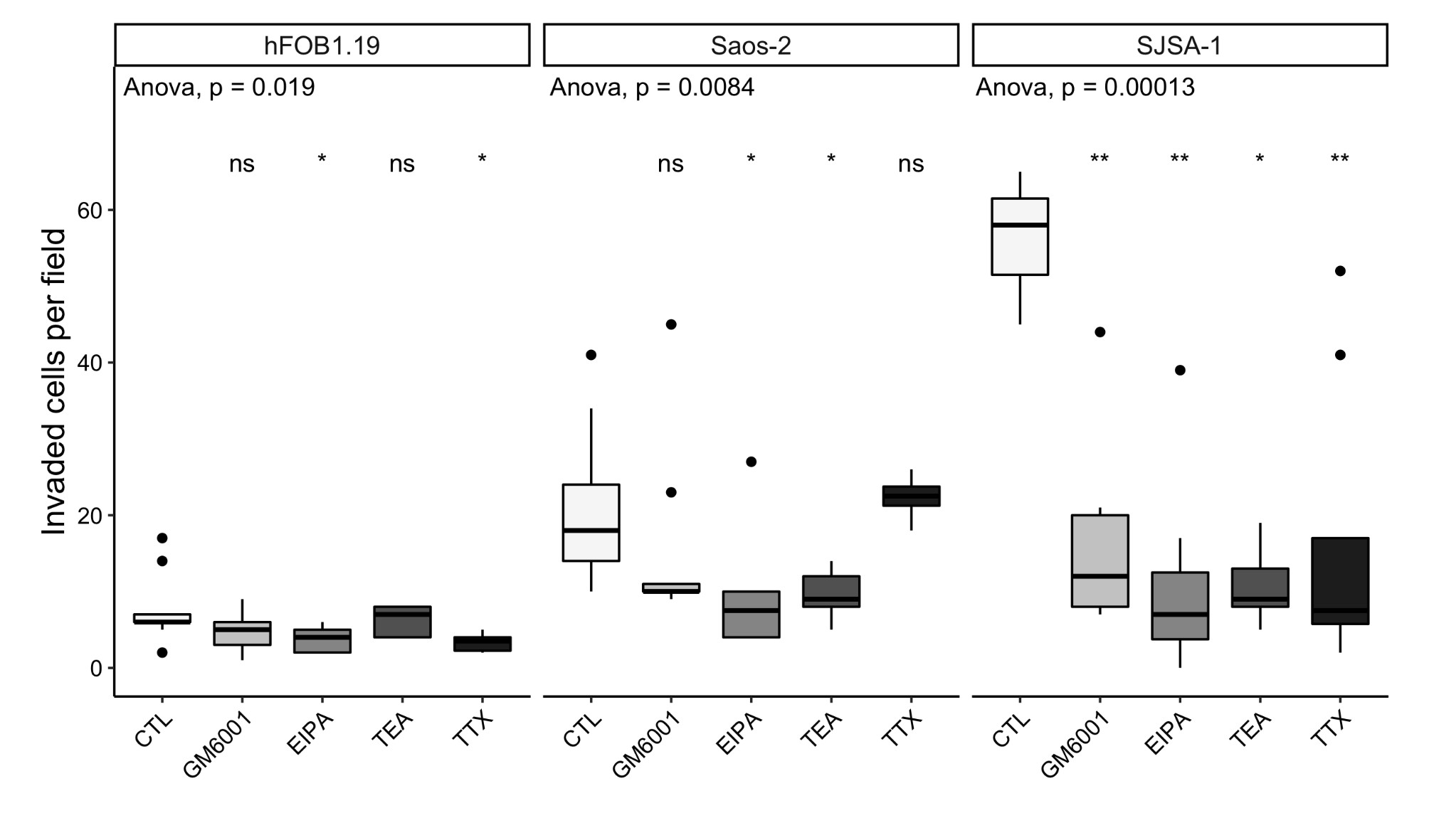


**Figure S5.** Quantification of invasive cells following various pharmacological treatments. This analysis was conducted based on data obtained from three independent experiments. ANOVA was employed to evaluate disparities among the treatment groups. Significance levels are represented as follows: ns (not significant, p > 0.05), * (p ≤ 0.05), ** (p ≤ 0.01), *** (p ≤ 0.001), and **** (p ≤ 0.0001).


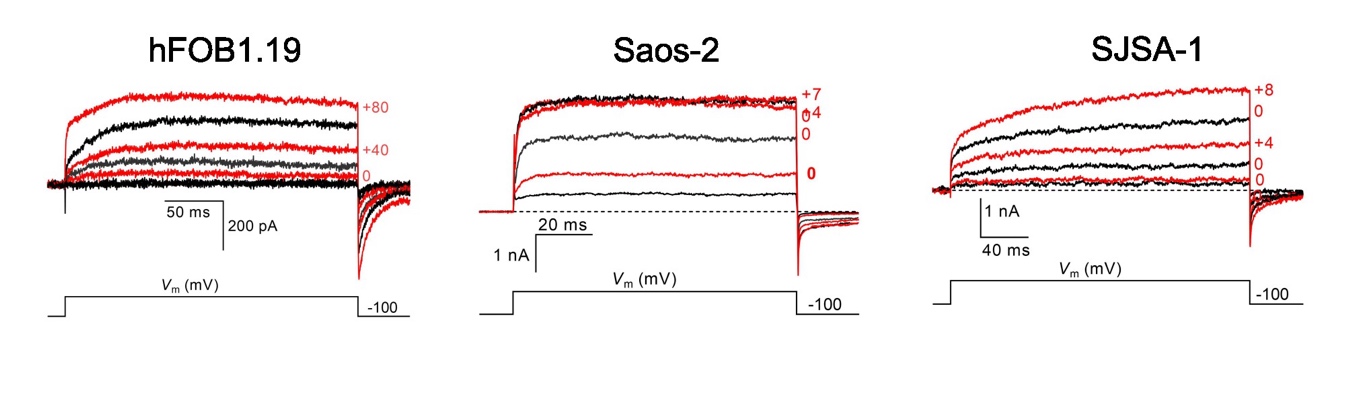


**Figure S6.** Functional expression of K_V_s in osteosarcoma. Representative currents generated by K_V_s were obtained in response to depolarizing pulses from -70 to +100 mV. For clarity purposes only recordings at -20, 0, +20, +40, +60, and +70 (SAOS2) or +80 (hFOB1.19 and SJSA1) are shown. Note the characteristic kinetics of K_V_s, where activation occurs followed by deactivation and channel closure, while inactivation is practically absent.


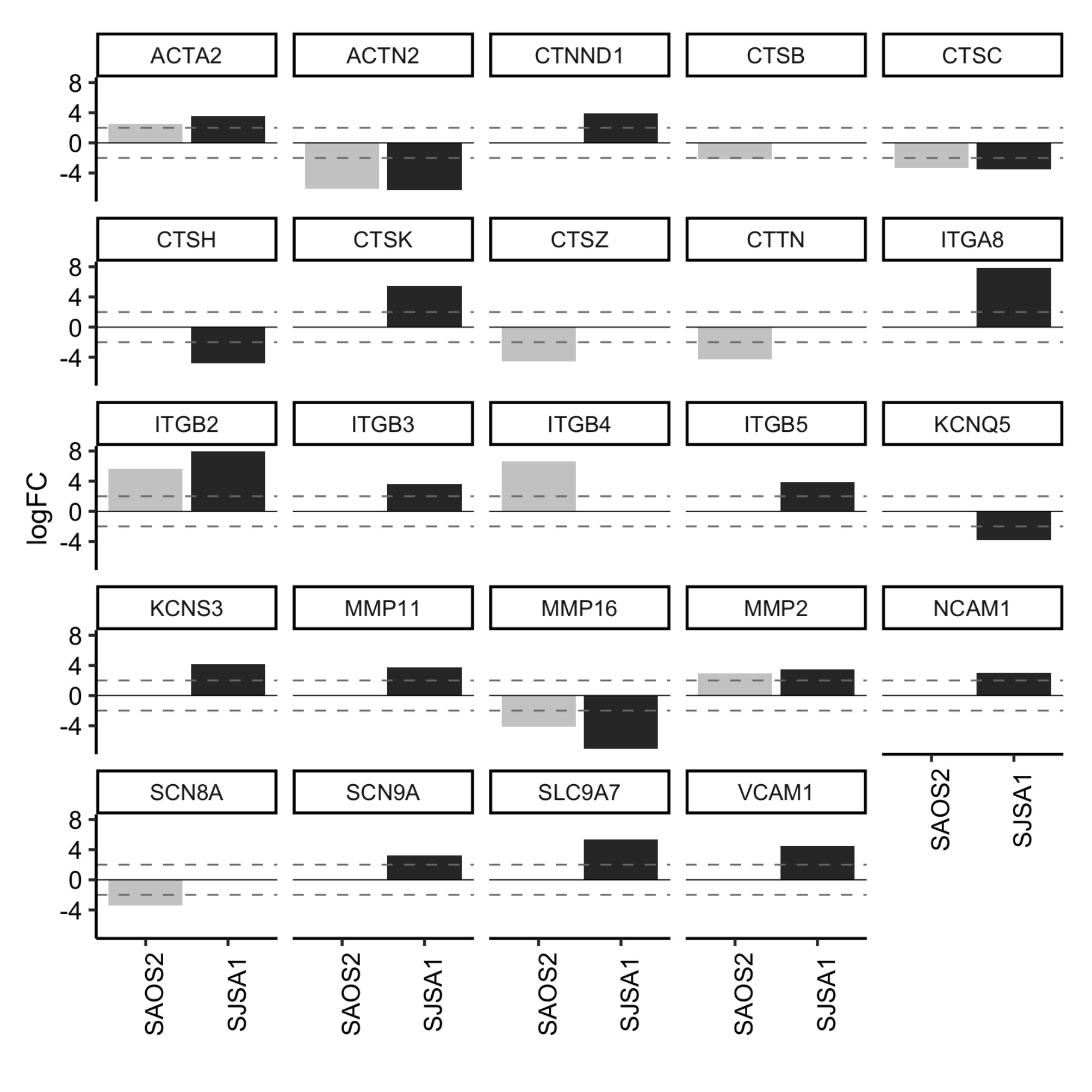


**Figure S7.** Osteosarcoma DEGs that have been reported in cancer invasion and metastasis. Dashed lines mark the LogFC threshold.
