# Supplementary material for: Biological Activity of Biomarkers Associated With Metastasis in Osteosarcoma Cell Lines
Source: Cancer Med. 2025 Mar 13;14(6):e70391. doi: 10.1002/cam4.70391 (PMC11904427; doi:10.1002/cam4.70391)
Supplement: Supplementary file 2 — Figure S1. [file CAM4-14-e70391-s002.docx]

**Supplementary Table S1.** RNA-seq aligment statistics. These statistics offer insights into the quality and quantity of RNA sequencing data, providing valuable information for downstream analysis and interpretation. ¶ Human genome reference: GRCh38p12.

| Sample ID | Total number of sequenced reads | Total number of uniquely mapped reads¶ | Total number of multi mapped reads¶ | RNA integrity number (RIN) | Ratio of all reads aligned to rRNA regions to total uniquely mapped reads (rRNA rate) | Total number of detected transcripts with reads ≥1 |
| --- | --- | --- | --- | --- | --- | --- |
| hFOB1.19 _1 | 21,728,108 | 7,708,667 | 19,140,617 | 8.1 | 0.032 | 29751 |
| hFOB1.19 _2 | 27,031,124 | 9,741,705 | 23,623,461 | 8.5 | 0.033 | 30140 |
| SAOS2_1 | 21,970,629 | 7,673,568 | 18,823,099 | 8.6 | 0.032 | 30116 |
| SAOS2_2 | 18,872,488 | 6,335,253 | 15,133,582 | 8 | 0.044 | 27845 |
| SJSA1_1 | 25,732,718 | 9,052,062 | 15,133,582 | 9 | 0.034 | 29768 |
| SJSA1_2 | 24,500,002 | 8,811,109 | 15,133,582 | 8.2 | 0.034 | 29856 |

**Supplementary Table S2.** Sequences of primers and TaqMan probes used for amplifying Na_V_s subunits

| **Subunit** |  | **Sequence (5’ → 3’)** | **Size (nt)** | **Tm** | **Amplicon** |
| --- | --- | --- | --- | --- | --- |
| PSMB2 (NM_002794.4 | Forward: | 5’-GAC TAT GTT CTT GTC GCC TCC-3’ | 21 | 58.46 | 101 |
|  | Reverse: | 5’-CAG CCT CTC CAA CAC ACA GG-3’ | 20 | 60.60 |  |
|  | Probe: | 5’-[FAM]-TCC GAC CGG GTG GCC GCC AGC A-[BHQ1]-3’ | 22 | 68.49 |  |
| β1-a (NM_001037.4) | Forward: | 5’-GTC TAC CGC CTG CTC TTC TTC-3’ | 21 | 60.47 | 101 |
|  | Reverse: | 5’-GAT GCC ATG TCT CTG TTG GC-3’ | 20 | 59.26 |  |
|  | Probe: | 5’-[FAM]-ACG AGC ACA ACA CCA GCG TCG TCA A-[BHQ1]-3’ | 25 | 69.13 |  |
| β1-b (NM_199037.4) | Forward: | 5’-CTC GCT GCA ATT TCA GTA TTG C-3’ | 23 | 58.90 | 114 |
|  | Reverse: | 5’-GTT CTC ACC ACC ATG ACC CGT-3’ | 21 | 62.32 |  |
|  | Probe: | 5’-[FAM]-TGA GCC ATG CCA TTT CCA TAG GTG GAG-[BHQ1]-3’ | 27 | 66.70 |  |
| β1-c (NM_001321605.1) | Forward: | 5’-CAG CGT TAG CTA CAG AGT GC-3’ | 20 | 58.73 | 101 |
|  | Reverse: | 5’-CAT ACA CGG CCT CGG TCT C-3’ | 19 | 59.93 |  |
|  | Probe: | 5’-[FAM]-AAG GAG GTG CCA GTG TCC TCA GCC T-[BHQ1]-3’ | 25 | 69.31 |  |
| β2 (NM_004588.4) | Forward: | 5’-GAA CCC CCG ACT TCG TAT CTC-3’ | 21 | 59.93 | 78 |
|  | Reverse: | 5’-CAG AGC ATG GCA GGT TTC TC-3’ | 21 | 58.91 |  |
|  | Probe: | 5’-[FAM]-ACC CTG CAC CAA GAG TGA CCC ACT C-[BHQ1]-3’ | 26 | 68.19 |  |
| β3-1 (NM_018400.3) | Forward: | 5’-GTC CTT GAC CGA GGG AAT CTC-3’ | 20 | 59.86 | 103 |
|  | Reverse: | 5’-GAT AAG CAC GAG AGA AGC CAG-3’ | 21 | 58.53 |  |
|  | Probe: | 5’-[FAM]-AGC CCC AGA AGA TGC CTG CCT TCA ATA-[BHQ1]-3’ | 27 | 67.86 |  |
| β3-2 (NM_001040151.1) | Forward: | 5’-CGA GAG CCT TGG AAG CCG-3’ | 18 | 60.51 | 107 |
|  | Reverse: | 5’-ACA CAG GGA AGC AGA CAC TG-3’ | 20 | 59.89 |  |
|  | Probe: | 5’-[FAM]-AGC CCC AGA AGA TGC CTG CCT TCA ATA-[BHQ1]-3’ | 30 | 67.86 |  |
| β4-1 (NM_174934.3) | Forward: | 5’-CAG GGT GGT TCA TTT CCC AG-3’ | 20 | 58.45 | 89 |
|  | Reverse: | 5’-GAA ACC CAA GGA CCC CCT C-3’ | 19 | 59.62 |  |
|  | Probe: | 5’-[FAM]-TCA AGG GCC TGC CTT CGC CTC AG-[BHQ1]-3’ | 23 | 68.62 |  |
| β4-2 (NM_001142348.1) | Forward: | 5’-CAC TGG GCT TTT GGT GGA AGA-3’ | 21 | 60.75 | 102 |
|  | Reverse: | 5’-CTT GAT CAG CAG GAT GAG GAT GA-3’ | 23 | 59.93 |  |
|  | Probe: | 5’-[FAM]-AGT GAC ACT CAT CAT CCT GGC TGT CGT-[BHQ1]-3’ | 27 | 67.45 |  |
| β4-3 (NM_001142349.1) | Forward: | 5’-GCT CCT TAC ACA GGC CTC TTC-3’ | 21 | 60.41 | 125 |
|  | Reverse: | 5’-CAG CTG GAG AAG GTG CAG G-3’ | 19 | 60.38 |  |
|  | Probe: | 5’-[FAM]-TGT CTG TGG GAA AGG CCA CCG ACA T-[BHQ1]-3’ | 30 | 68.21 |  |
| NaV1.5 (NM_198056.2) | Forward: | 5’-GAA CAG CAC TGT GGA CTG CA-3’ | 20 | 60.39 | 79 |
|  | Reverse: | 5’-GTG GCT TCC TGG GGA TGT G-3’ | 19 | 60.38 |  |
|  | Probe: | 5’-[FAM]-TCT CAT TAC TGG GGG CAG GCG ACC-[BHQ1]-3’ | 30 | 68.17 |  |
| NaV1.6-1 (NM_014191.3) | Forward: | 5’-GTG GTG GCC ATG GCT TAT GA-3’ | 19 | 60.40 | 121 |
|  | Reverse: | 5’-CAG CCT GTG CCT CTT CCT G-3’ | 19 | 60.38 |  |
|  | Probe: | 5’-[FAM]-ACA GAA TCA GGC AAC ACT GGA GGA GG-[BHQ1]-3’ | 30 | 66.29 |  |
| NaV1.6-2 (NM_001177984.2) | Forward: | 5’-GTG GCT GTG GTG GTG AAT G-3’ | 19 | 59.04 | 120 |
|  | Reverse: | 5’-CTT TCC CGC AAA CAA GTT AAC TCC-3’ | 24 | 60.79 |  |
|  | Probe: | 5’-TGT GCT GCT GGT GTG TCT CAT CTT CTG-3’ | 30 | 66.80 |  |
| NaV1.7 (NM_002977.3) | Forward: | 5’-GGC TGC TAC CTC CAC GG-3’ | 17 | 59.43 | 97 |
|  | Reverse: | 5’-GGA ATT GGA AAG CCG ACA GC-3’ | 20 | 59.83 |  |
|  | Probe: | 5’-[FAM]-AGG AGG GGC GCA GTC TGC TTG CA-[BHQ1]-3’ | 25 | 70.00 |  |

**Supplementary Table S3.** Information on network topology and parameters, including the number of nodes (N), edges (Edges), average degree (<k>), clustering coefficient (<c>), diameter (D), and hub value (Hub_value), which represents the degree value cutoff used for identifying hubs in the network.

| **Network** | **N** | **Edges** | **<k>** | **<c>** | **D** | **Hub_value** | **Hubs** |
| --- | --- | --- | --- | --- | --- | --- | --- |
| hFOB1.19 vs Saos-2 | 138 | 679 | 4.92 | 0.236 | 6 | 18.479 | COL6A2, COL6A3, KIT, SERPINE1, COL3A1, ICAM1, IL1B, COL4A1, COL4A2, CXCL8, ITGA6, ITGA7, ITGB4, TP53, FGF2, ITGA2, MMP2, COL1A1, IL6, TGFB1, SRC, CD44, FN1 |
| hFOB1.19 vs SJSA-1 | 122 | 510 | 4.18 | 0.230 | 7 | 14.929 | ITGA8, MMP2, CASP3, MYC, CXCL1, ITGB5, ITGB2, CCL5, MET, CSF2, CSF3, ITGB3, PDGFRA, CXCR4, PDGFRB, CXCL12, FGF2, IL1A, STAT1, CXCL8, CD44, IL1B, IL6 |
| Saos-2 vs SJSA-1 | 123 | 474 | 3.854 | 0.184 | 7 | 14.591 | SDC1, LAMB1, COL3A1, THBS2, COL6A3, TGFB1, COL4A1, COL6A1, COL6A2, FGF2, ITGA6, ITGA11, ITGA7, COL4A2, ITGA8, ITGB4, COL1A2, TP53, FN1, ITGB3 |
